# Classical and computed tomographic anatomical analyses in a not-so-cryptic *Alviniconcha* species complex from hydrothermal vents in the SW Pacific

**DOI:** 10.1186/s12983-020-00357-x

**Published:** 2020-05-07

**Authors:** Sven R. Laming, Stéphane Hourdez, Marie-Anne Cambon-Bonavita, Florence Pradillon

**Affiliations:** 1 Ifremer, Laboratoire Environnement Profond (REM/EEP/LEP), Plouzané, France; 2 Ifremer, Univ Brest, CNRS, UMR6197, Laboratoire de Microbiologie des Environnements Extrêmes (REM/EEP/LM2E), Plouzané, France; 3 Current address: LEME, CESAM - Centre for Environmental and Marine Studies, Department of Biology, Universidade de Aveiro, Santiago Campus, 3810-193 Aveiro, Portugal; 4 UMR 8222 CNRS-Sorbonne Université, Laboratoire d’écogéochimie des environnements benthiques (LECOB), Banyuls-sur-Mer, France

**Keywords:** Deep sea, Chemosymbiotic, Periostracum, Gastropod, Habitat partitioning, Computed tomography, Histology, Taxonomy, 3D model

## Abstract

The chemosymbiotic gastropod *Alviniconcha* (Provannidae), first described in 1988, is one of the most emblematic hydrothermal-vent taxa described from the Central Indian Ridge and the Southwest (SW) Pacific. Symbiotic bacteria found in the gill of *Alviniconcha* are thought to be their principal source of nutrition. In the SW Pacific, species distributions for *A. kojimai*, *A. boucheti* – and to a lesser extent *A. strummeri* – overlap. While *Alviniconcha* species do not appear to truly co-exist in these highly energetic but spatially limited habitats, certain species regularly co-occur within a single vent field and in rare instances, the same edifice. Past research suggests that SW-Pacific *Alviniconcha* species might aggregate around fluids with distinct geothermal profiles. These small-scale distribution patterns have been attributed to differences in their symbiont assemblages or host physiologies. However, little is known about the anatomy of most *Alviniconcha* species, beyond that detailed for the type species *Alviniconcha hessleri*, whose geographic range does not overlap with other congeners. In fact, species within this genus are currently described as cryptic, despite the absence of any comparative morphological studies to assess this. To test whether the genus is genuinely cryptic and identify any functional differences in host anatomy that might also mediate habitat partitioning in SW Pacific species, the current study examined the morphoanatomy of *A. kojimai*, *A. boucheti* and *A. strummeri* from the Fatu Kapa vent field, an area of hydrothermal activity recently discovered north of the Lau Basin near the Wallis and Futuna Islands and the only known example where all three species occur within adjacent vent fields. A combination of detailed dissections, histology and X-ray computed tomography demonstrate that *A. kojimai*, *A. strummeri* and *A. boucheti* are readily identifiable based on shell morphology and ornamentation alone, and therefore not truly cryptic. These traits provide a rapid and reliable means for species identification. However, aside from some subtle differences in radular morphology, these species of *Alviniconcha* exhibit conserved anatomical features, providing no evidence that functional host anatomy is implicated in habitat partitioning. This provides support for the current belief that host-species distributions are probably governed by symbiont-mediated physiological factors.

## Background

The chemosymbiotic gastropod genus *Alviniconcha* (Provannidae) is one of the most abundant, emblematic taxa of hydrothermal-vent communities described from the Central Indian Ridge and the SW Pacific, including the Mariana volcanic arc and the Mariana, Manus, North-Fiji and Lau back-arc basins [1]. This genus was erected in 1988, with a preliminary description of *Alviniconcha hessleri* Okutani & Ohta, 1988, based on specimens from the Mariana Back-arc Basin [2]. Soon after in 1993, an emended diagnosis of this species provided a detailed account of adult morphoanatomy and larval-shell characteristics for the first time [3], based on new *Alviniconcha* specimens collected from the vent sites “White Lady” (North-Fiji Basin) and “Vai LiIi” (Lau Basin, with additional juveniles from “Hine Hina”). In that paper, Warén and Ponder identified a high degree of intraspecific plasticity in larval-shell morphology and stated more generally that the species’ shell was “of little use for classification” ([3]: p. 56). The same year, another study analysing the genetic diversity of *Alviniconcha* specimens from the same sites presented strong evidence for the existence of multiple species [4]; unfortunately, material from the original Mariana Back-arc population was lacking, precluding any confirmation that *A. hessleri* was one of the species at these sites. Evidence for the existence of an *Alviniconcha* species complex has grown in the decades that have followed [5–8], as has our understanding of a genus-wide dependency upon chemosymbiosis for nutrition (e.g. [9–13]), expanding on earlier, pioneering research in this field [14, 15]. Robust molecular diagnoses based on concatenated marker gene analyses have recently been published for the remaining species: *A. adamantis*, *A. boucheti*, *A. kojimai*, *A. marisindica* and *A. strummeri* Johnson, Warén, Tunnicliffe, Van Dover, Wheat, Schultz & Vrijenhoek 2015, resulting in six distinct evolutionary lineages including that of *A. hessleri* [1]. An inherent outcome, however, is that species identification remains entirely dependent upon molecular analyses. Conflicting accounts may be found in the literature of the utility of morphological features in distinguishing *Alviniconcha* species. Denis and colleagues [4] commented on what they believed to be species-specific shell features as further evidence to support their hypothesis for the presence of multiple species in *Alviniconcha*; this was also touched upon, albeit briefly as a side note to a preliminary description of another provannid from the SW Pacific. Yet, several studies have stated the opposite, be it because of confounding phenotypic plasticity (inadvertently based on undescribed species rather than the species thought to be under investigation [3, 16]), or – as exemplified by the molecular diagnoses for *Alviniconcha* – because no distinguishing morphological traits could be confidently related to a given clade ([1], but also in [8]). Few studies have presented species morphoanatomy in much detail to support either argument ([9] presents gross anatomy, but in the context of a symbiosis-based paper) and none have employed an exhaustive, comparative approach.

**Fig. 1:**
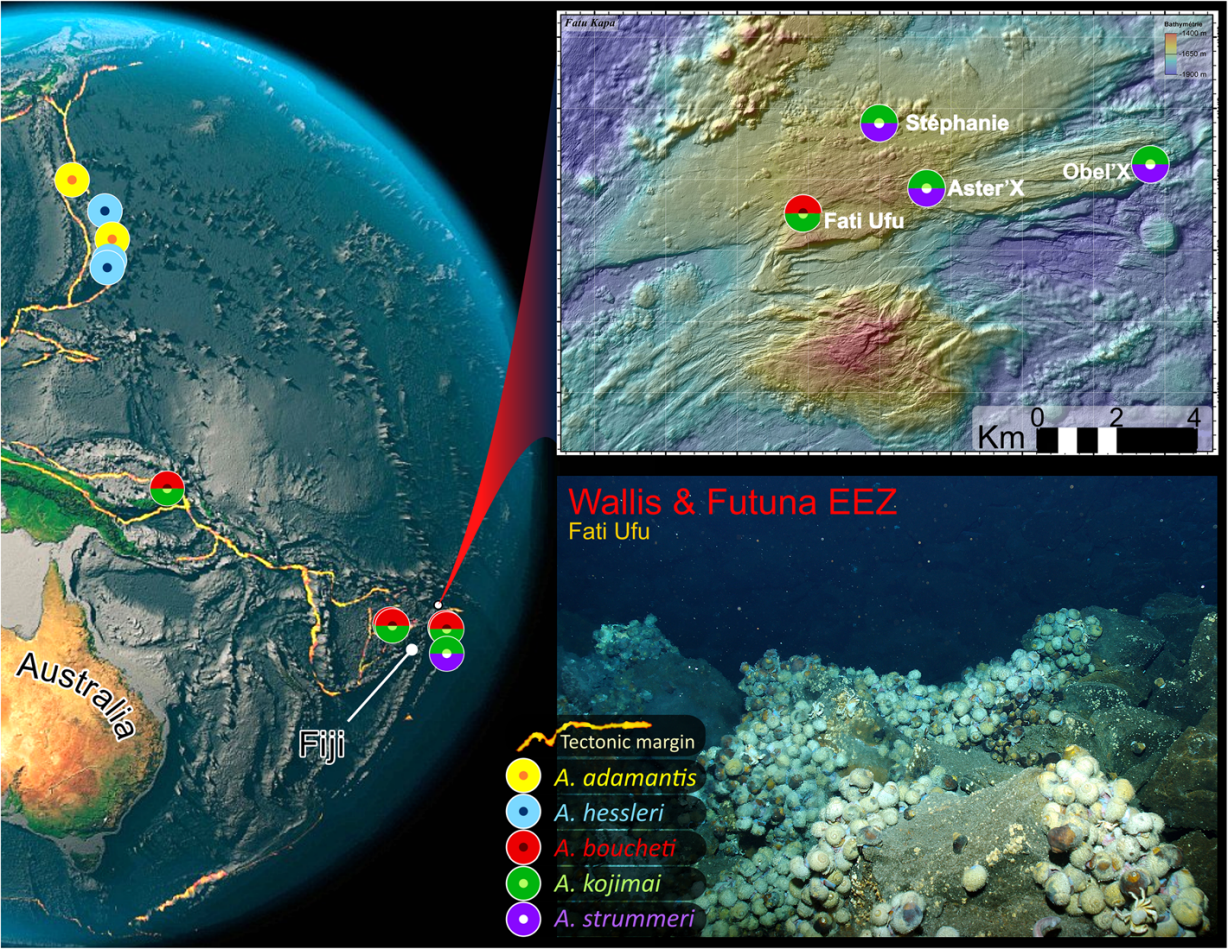
Map of SW-pacific region, *Alviniconcha* species records and Fatu Kapa vent-field study sites. Photo of habitat taken in northern area of Fati Ufu during cruise. The current distribution records for the five Pacific species indicate that only *A. kojimai* and *A. boucheti* are found together with any regularity. The distribution of the sixth species, *A. marisindica*, is restricted to sites on the Central Indian Ocean Ridge (not shown). Image credits: Globe *Amrita Carroll*; Fatu Kapa Map *Futuna 3 cruise, AUV AsterX*; Fati Ufu site photo *Futuna 3 cruise, HOV Nautile*

The molecular assignments of Johnson et al. [1] have nonetheless helped to clarify the distribution of the six species now described, all of which are obligate hydrothermal-vent fauna. Geographic ranges of the five described species from the Pacific abut one another at an oceanic-basin scale. Records of more than one *Alviniconcha* species over smaller spatial scales (< 100-km separation) are restricted to select regions in the SW Pacific [1]. Current understanding indicates that separate species do not form mixed colonies, though they have been recorded in different places on a single edifice [11]. *Alviniconcha kojimai* and *A. boucheti* regularly occur in proximity to one another throughout their broad species distributions in the Manus and North-Fiji Basins, sometimes within a single vent field at sites only 10–100 m apart. In the northern Lau Basin, the boundary of *A. boucheti*’s geographic range, they are both recorded from shared edifices but in these instances, one species is always overwhelmingly dominant (*A. boucheti* dominates at Kilo Moana KM-2, all Tow Cam vent sites, and ABE-1, while *A. kojimai* dominates at ABE-2 and -4). *Alviniconcha kojimai* records continue farther south in the Lau Basin where, at Tu’i Malila vent sites, it occurs on adjacent or shared edifices in lower numbers with the dominant species *A. strummeri* [11]*,* the latter being an otherwise-rare member of the genus with a geographic distribution restricted to the southern Lau basin and predominantly at Tu’i Malila (at time of writing, see Fig. 1).

*Alviniconcha* form dense aggregations around the bases and walls of active chimneys [17] which issue chemically reduced fluids that provide both energy and carbon for chemosynthesis. High thermal tolerances [18] and branchial, chemosynthetic, bacterial symbioses [9–15] which facilitate high potential growth rates [18] and probably represent the host’s primary source of nutrition [19], enable *Alviniconcha* to take direct advantage of these resources. Residing in the host’s hypertrophied ctenidium, these intracellular – but occasionally intracytoplasmic or intravacuolar [15] – symbionts may also provide a secondary fluid-detoxification role. Denis et al. [4] were the first to identify a potential correlation between the spatially discrete – but often proximate – distribution patterns observed for SW Pacific *Alviniconcha* species and local-scale geochemistry. Research since has suggested that these small-scale differences in host-species distributions across sites with marked differences in fluid composition, could relate to the distinct bacterial symbiont assemblages that each host species possesses [10, 11]. Most *Alviniconcha* species, including *A. kojimai* and *A. strummeri,* host Gammaproteobacteria-dominated symbioses that are closely related to known chemoautotrophic [9–11, 14, 15], sulphur-oxidising bacteria that predominantly use the Calvin–Benson–Bassham cycle for carbon fixation [20]. In the remaining two species, *A. boucheti* and *A. marisindica*, symbioses are dominated by sulphur-oxidising bacteria from a separate phylum, the Campylobacterota [9, 10] – formerly known as the Epsilonproteobacteria [21, 22] – that also utilise H_2_-oxidation [23–25] under high-H_2_ concentrations (several mM, [25]) and likely fix carbon through the Reverse Tricarboxylic Acid cycle [20]. Thus, one hypothesis that might explain the small-scale habitat partitioning observed in SW Pacific *Alviniconcha* species, is that optimal conditions for chemosynthesis differ for each host species, as a function of the unique metabolic capabilities or physiological requirements of their symbiont assemblages. Interestingly, phylogenetic analyses of recently published symbiont genomes from SW pacific *Alviniconcha* species indicate that chemoautotrophic function in symbiotic bacteria is similar irrespective of host species [24], suggesting differences relating to gene expression (e.g. [25]) and regulation and/or differences in host physiology may mediate habitat partitioning.

However, knowledge gaps concerning *Alviniconcha* anatomy – documented in the type species only – undermine our capacity to validate this hypothesis. It is not yet known whether there are adaptive functional traits specific to each host species that might also be implicated in the distribution of SW Pacific *Alviniconcha* species. More fundamentally, such gaps impede real-time species identification. Currently, the discrimination of *Alviniconcha* to species level requires a molecular approach involving DNA extraction, amplification and sequencing. This is typically performed after sample collection and processing and therefore cannot inform experimental design and sampling strategies a priori or alterations frequently necessary due to the logistics of sampling deep-sea environments. Anatomical studies can provide great insight regarding the evolutionary adaptations that organisms have developed to survive in their environment. For chemosymbiotic species, accommodating bacterial partners at high densities typically requires the alteration of an existing organ, such as tissue hypertrophy and differentiation in gills (many examples in [26]) or the oesophageal gland [27, 28], or the emergence of a novel specialised organ such as the siboglinid trophosome [29], dedicated to this purpose. Diverse adaptations such as these are typically discovered through detailed histological and anatomical analyses, offering fascinating new ecological insights and evolutionary perspectives. However, due to a continued focus on symbiotic tissues in most *Alviniconcha* species, accounts of remaining host anatomy are limited and sparse in detail. To address this, the current study documents the comparative morphoanatomy of members of this genus from the Fatu Kapa vent field, an area of hydrothermal activity recently discovered north of the Lau Basin near the Wallis and Futuna Islands. This region is not only highly active, geothermally [30] and rich in metals [31–33] but also a rare instance where *A. kojimai*, *A. boucheti* and *A. strummeri* are documented at sites only a few kilometres apart: an ideal scenario for examining comparative host biology. The current study employs a comparative anatomical approach using morphology, microscopy and computed tomography (CT) to assess whether: 1) the three congeneric gastropod species *A. kojimai*, *A. boucheti* and *A. strummeri*, occurring in close proximity, are actually cryptic as documented, and; 2) whether there are species-specific aspects of anatomy, outside of symbioses, that might be driving hypothesised habitat partitioning.

## Results

### Species distribution and identification

Although species distribution data is limited for the current samples collected in the Wallis and Futuna volcanic region, we know that at least some specimens of *A. kojimai* and *A. strummeri* were taken from single gastropod patches, recorded for the first time (co-occurring less than a metre apart). Unfortunately, data on the proximity of *A. boucheti* and *A. kojimai* when co-occurring are unavailable, as specimens were a pooled collection from multiple sampling locations during a dive where both species were found (though still only several metres apart).

Nucleotide sequence data from 106 specimens targeting the 1228 bp fragment of the mitochondrial gene, cytochrome-c oxidase subunit I (COI), have been deposited in GenBank (see Table 1). Anatomical descriptions detailed below are listed by functional system. For each subsection, features applicable to all three study species (exemplified at times by figures featuring a single species) are described first, followed by features identified as specific to individual species, where applicable (summarised in Table 2). Note that interactive anatomical models are available for CT-based visualisations performed on *A. kojimai* and *A. strummeri* specimens. The interactive model for *A. kojimai* is embedded in Additional file 1. The interactive model for *A. strummeri* is embedded in Additional file 2.

**Table 1:** GenBank Accession numbers by species and site for the three *Alviniconcha* spp.

		***A. kojimai*** (66)	***A. strummeri*** (8)	***A. boucheti*** (32)
Vent sites	**Stéphanie** (5)	MT010417 – MT010420 (4)	MT010483 (1)	-
	**Obel’x** (28)	MT010421 – MT010444 (24)	MT010484 – MT010487 (4)	-
	**Aster’x** (24)	MT010445 – MT010465 (21)	MT010488 – MT010490 (3)	-
	**Fati Ufu** (49)	MT010466 – MT010482 (17)	-	MT010491 – MT010522 (32)

**Table 2:**
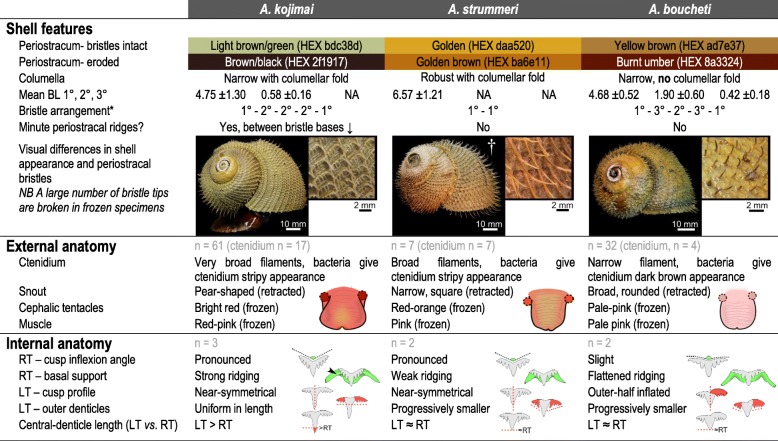
Summary of distinguishing characteristics among the three *Alviniconcha* spp.

Species were readily distinguishable based on periostraca. *BL* bristle length, *RT* rachidian, *LT* Lateral teeth. Mean BL calculated based on lengths from 10 individuals of *A. kojimai* and *A*. *boucheti* and 5 individuals of *A*. *strummeri* (based on three replicates ea.). *A fourth 2° bristle can occur in *A. kojimai,* very occasionally and sporadically*.* † Photo is of an alcohol-fixed specimen (other photos from frozen specimens)

### Shell morphology and ornamentation

#### General observations

**Fig. 2:**
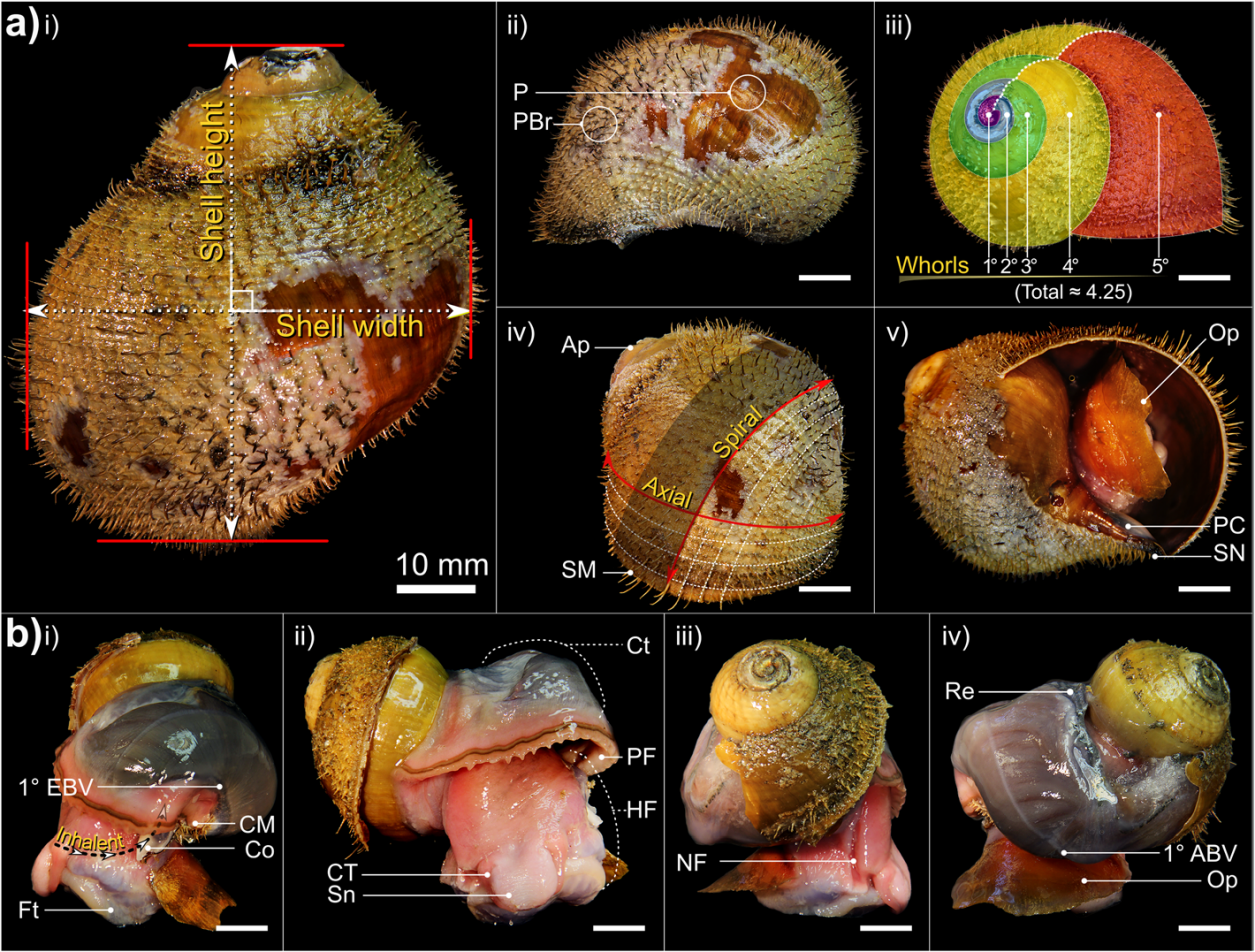
Aspects of gross morphology and explanation of certain malacological terms. Example is *A. boucheti*: **a** i) abapertural view with shell dimensions, ii) comparison of bare (PBr) and adorned periostracum (P) in lateral-left view, iii) spiral distance from the apex, measured in whorls, iv) spiral and axial lines in relation to the apex (Ap) and shell margin (SM) and v) apertural view with only a slight parietal callus (PC) and siphonal notch (SN) present; **b** lateral-left, anterior, lateral-right and near-posterior views of a specimen with a whorl removed. An equivalent micrograph plate for *A. kojimai* may be found in Additional file 3. NB. the ctenidium (Ct) in *A. boucheti* is darker in appearance than that of *A. kojimai* and *A. strummeri*. Additional abbreviations: 1° ABV Primary afferent branchial vessel; 1° EBV Primary efferent branchial vessel; CM Columellar muscle; Co Columella; CT Cephalic tentacle; Ft Foot; HF Head-foot; NF Neck furrow; Op Operculum; PF pallial fringe; Re Rectum; Sn Snout

Shells are dextral, hirsute and flexible with little mineralised support, except the columella, which is calcareous and robust. Shell outlines are subglobular (e.g. Fig. 2, Additional file 3). The number of whorls is dependent on shell height (SH) with maximum of 4.5–5 whorls at SH > 70 mm (Fig. 2a-iii). The larval protoconch is invariably absent and spires are rounded (presumably due to dissolution and mechanical erosion), revealing a thick multi-layered periostracum and argenate ostracum. The body whorl is greatly enlarged at around ~ 70–80% SH (Fig. 2a, Additional file 3a)*.* The aperture is large, sub-oval with a slight parietal callus. The columella possesses a small siphonal notch only (Fig. 2a, Additional file 3a). All three study species possess a dense, regular, spiral arrangement of non-calcareous periostracal bristles that adorn the latter whorls, being absent on the apex. In all species, bristle lengths (BL) within a single spiral row were approximately congruent, with slight decreases in length spirally, with decreasing whorl width. Within each BL class (see next section), medial bristles were also slightly longer than those nearest sutures, the junctures between adjacent whorls (e.g. Fig. 2a, v). However, BL displayed very striking, species-specific arrangements when viewed axially (i.e. along co-marginal bristle lines, discussed below).

#### Species-specific shell characteristics

**Fig. 3:**
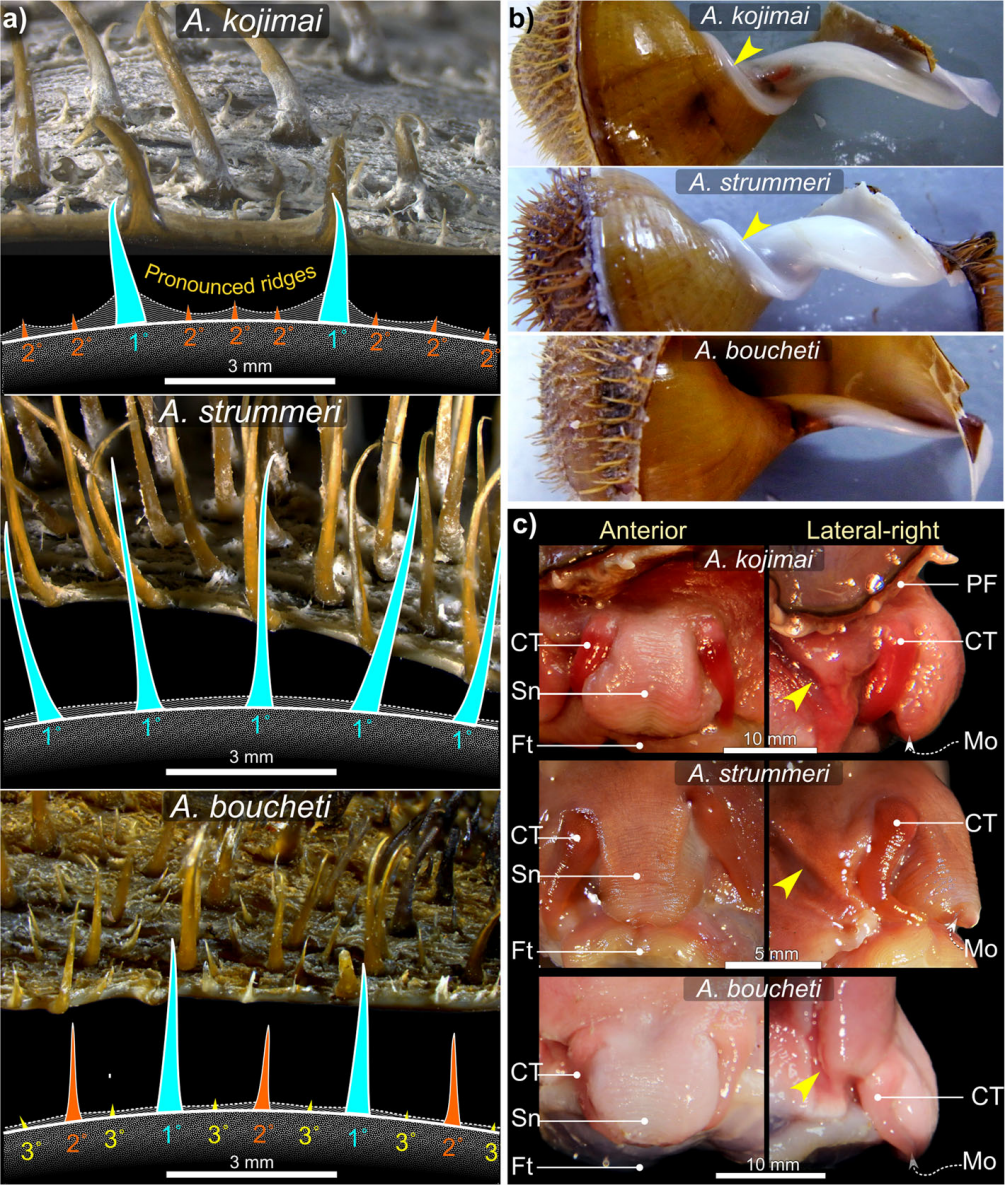
Distinguishing morphoanatomical characteristics of the shell and head-foot . External, species-specific, morphological features include: **a** the lengths and arrangement of primary (1°), secondary (2°) and tertiary (3°) periostracal bristles; **b** the architecture and diameter of the columella where arrowheads indicate the columellar fold (absent in *A. boucheti*) and; **c** head-foot coloration (arrowheads indicate neck furrows). Abbreviations: CT Cephalic tentacles; Ft Foot; Mo Mouth; PF Pallial fringe; Sn Snout

Mean SH for *A. kojimai*, *A. strummeri* and *A. boucheti* were SH 60.08 mm ± σ 14.2 (SH_range_ = 37.8–89.1 mm, *n* = 44), 55.33 mm ± σ 10.2 (SH_range_ = 46.1–53.7 mm, *n* = 4) and 65.19 mm ± σ 5.2 (SH_range_ = 57.1–77.6 mm, *n* = 14) respectively, measured in a random subset of individuals. In specimens from the current study site, periostraca were typically laurel-green to light brown in *A. kojimai* and *A. strummeri* (due in part to a thin white layer of bacterial flocculent) and burnt-umber brown in *A. boucheti* (Table 2). In *A. kojimai* and *A. strummeri*, a columellar fold arises internally in the first (apical) whorl and descends as an adapical spiral as far as 0.5 whorls back from the aperture opening (Fig. 3b, Additional file 4). This fold is particularly pronounced in *A. strummeri* in which the columella is notably thicker (relative to shell width) than that of *A. kojimai* and *A. boucheti* (Additional file 4). A columellar fold is absent in *A. boucheti* (Fig. 3b, Additional file 4). The arrangement and lengths of periostracal bristles follow species-specific patterns (observed in ninety specimens, Fig. 3a). In *A. boucheti* and *A. kojimai,* BL alternated markedly in length axially (Table 2, Fig. 3a). Three discrete BL were identifiable in *A. boucheti* (− 80 °C, *n* = 29): long primary (1°), shorter secondary (2°, at ~ 50% of primary BL) and much shorter tertiary bristles (3°, at < 10% of primary BL). Between each spiral row of 1° bristles were three rows of the shorter bristles, arranged in a − 3°-2°-3°- sequence (Fig. 3). In *A*. *kojimai* (− 80 °C, *n* = 52; 4%-formalin-fixed, *n* = 2), two to three markedly different BL were identifiable. Longer (1°) bristles were easily identifiable. Between each spiral row of 1° bristles were three rows (occasionally four) of much shorter 2° bristles (5–10% of primary BL). The 2° bristles were generally congruent but in a minority of individuals, medial 2° bristles were almost double the length of other 2°, thus superficially resembling the bristle arrangement of *A*. *boucheti*. However, a lattice-like network of minute spiral and axial ridges connects bristles to one another at their bases in *A. kojimai* (Additional file 3), a feature not evident in the other two study species (Table 1), though this feature can be obscured by bacterial flocculent (as in Fig. 3a). By employing this characteristic, ambiguous *A*. *kojimai* specimens were usually distinguishable from *A. boucheti*. Contrary to the other species, bristles in *A*. *strummeri* were universally congruent with no obvious axial differences in BL (except general trends described in 4.2.1) and comparatively longer than the longest (1°) BL of the other study species, with greater mean BL in adults of equivalent SH (Table 1, Fig. 3).

### External morphoanatomy

#### General observations

In a 4-whorl shelled specimen (~ 50–60 mm SH), soft-body occupies the last (i.e. most recently formed) 2–2.5 whorls. The muscular head-foot occupies the last 0.1–0.25 whorls, depending on the degree of extension beyond the aperture. When preserved, the snout is short with tight, transverse epidermal folds both dorsally and ventrally, suggesting some capacity for elongation. Two cephalic tentacles, lacking basal eye spots, flank the snout (~ 2 x snout length). The ventral underside of the snout is flush with the anterior flank of the propodium and whitish-pink in colour, with a small distal mouth concealed from view dorsally. In chemically preserved specimens, the mouth was tightly contracted and thus difficult to discern. The foot is large, wide and fleshy; the sole possesses translucent pale blue-purple bulges within which are the pedal blood sinuses (Additional file 3v, the anterior-most, medial propodal bulge being considerably larger and ovoid: 30% foot-width laterally). On both sides of the foot, a lateral epipodial fold extends anteriorly from beneath the attachment region for the operculum, terminating below the cephalic tentacles. A deep cephalopedal channel, or neck furrow (Fig. 2b-iii and Additional file 3, ‘NF’), demarcated by parallel epidermal folds, descends from within the pallial cavity down the right flank of the head-foot, becoming wider and less defined as it terminates below the right cephalic tentacle on the anterior-most point of the epipodial fold. In some specimens, the neck furrow possesses another narrow, central fold (Additional file 5a), thus creating twinned channels, which ultimately diverge below the right cephalic tentacle. The operculum is attached to the metapodium along the posterior-most edge of the epipodial folds and is similar in all three species: ovo-quadrate, thickened proximally, with an irregular, curved distal margin. The nucleus is not evident.

**Fig. 4:**
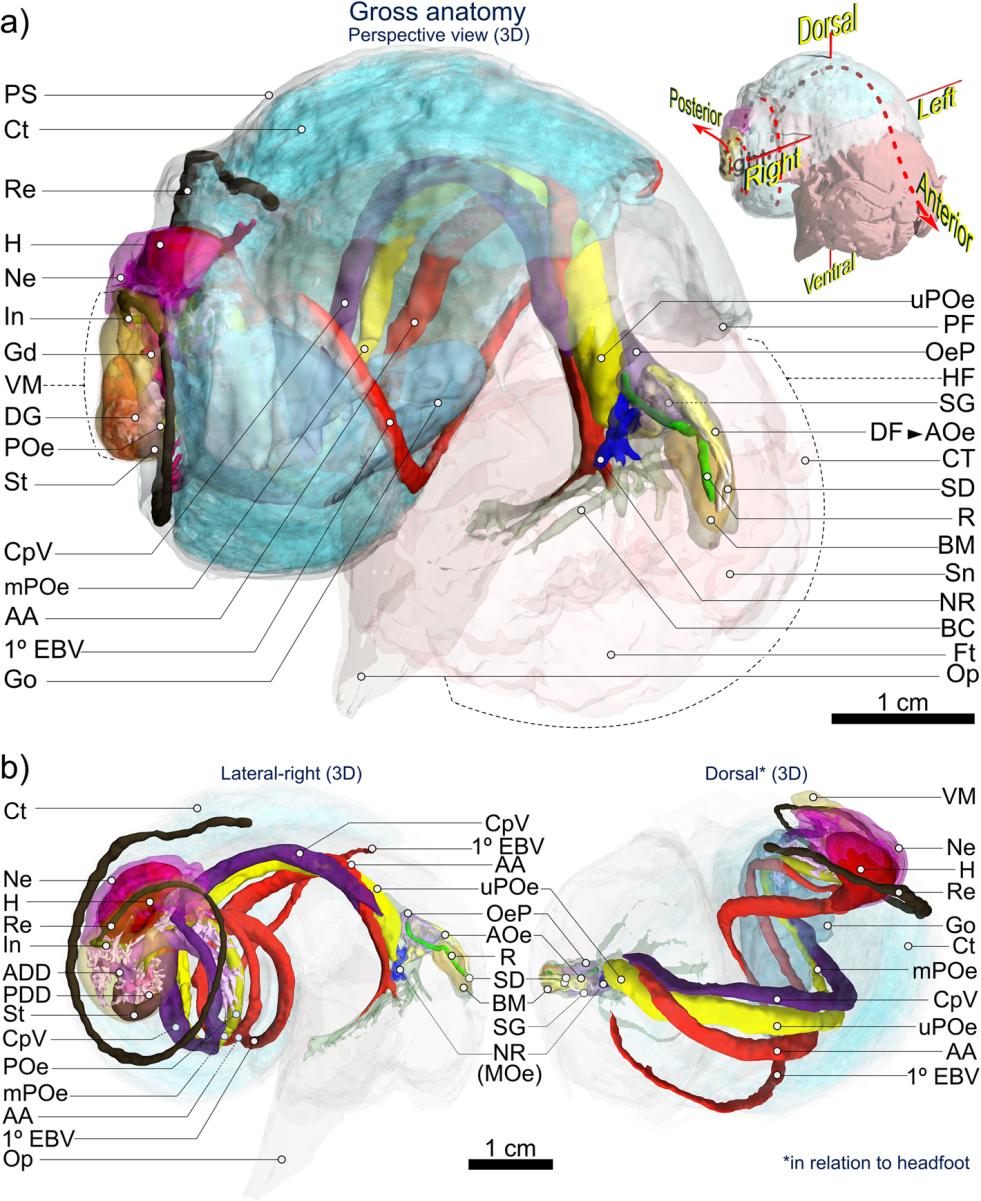
3D visualisation of *Alviniconcha* gross anatomy based on computed tomography (CT). Images are from the *A. kojimai* specimen scanned using computed tomography. A more detailed interactive model of this specimen is embedded in Supplementary figure 1 and a second interactive model for *A. strummeri* is included in Supplementary figure 2. Note that for clarity purposes, some tissues and features present in the interactive model, are not included in the above 3D visualisation (but see Figs. 6 and 8). Abbreviations: 1° EBV Primary efferent branchial vessel; AA Anterior aorta; ADD Anterior digestive duct; AOe Anterior oesophagus; BC Buccal cavity; BM Buccal mass; CpV Cephalopedal Vein; Ct Ctenidium; CT Cephalic tentacles; DF Dorsal fold; DG Digestive gland; Ft Foot; Gd Gonoduct; Go Gonad; H Heart; HF Head-foot; In Intestine; MOe Mid-oesophagus; mPOe Mid-posterior oesophagus; Ne Nephridium; NR Oesophageal nerve ring; OeP Oesophageal pouches; Op Operculum; PDD Posterior digestive duct; PF Pallial fringe; POe Lower posterior oesophagus; PS Pallial skirt; R Radula; Re Rectum; SD Salivary ducts; SG Salivary glands; Sn Snout; St Stomach; uPOe Upper posterior oesophagus; VM Visceral mass. Figure is of 3D renderings with exaggerated perspective, thus scales are approximate

The anterior pallial margin is muscular, possessing a large dark-coloured co-marginal vessel and a distal anterior fold, from which project numerous, stout, tapering papillae, each with a medial, dorsal fold. Papillae are longest on the right of the pallial margin, small and indistinct on the far left. On the right, the final few millimetres of pallial margin deflect inwards, attaching to the head-foot laterally, posterior-ventral to the neck furrow’s steepest point of descent. On the left, the pallial margin is attached via a thickening, siphon-shaped fold of muscle (inhalant region, Fig. 2b, i), adhered to the shell via the columellar muscle ~ 0.5 whorls back (itself continuing ~ 0.75 whorls farther). The pallial cavity extends 1–1.25 whorls back from the pallial margin (~ 0.1 whorls behind the outer apertural lip), decreasing rapidly in volume posteriorly as the cavity narrows and loses height (*A. boucheti*, Fig. 2b; *A. kojimai,* Fig. 4, Additional files 1 and 2; *A. strummeri,* Additional file 2). Viewed dorsally, the ctenidium (extending left-to-right) and rectum (far-right) are visible through the pallial epithelium, which is delicate and translucent (e.g. Fig. 2). On the right side of the pallial cavity in areas where ctenidium is absent, the pink (anterior) or purple-black (posterior) epithelial surface of the underlying cephalopedal haemocoel can be seen.

**Fig. 5:**
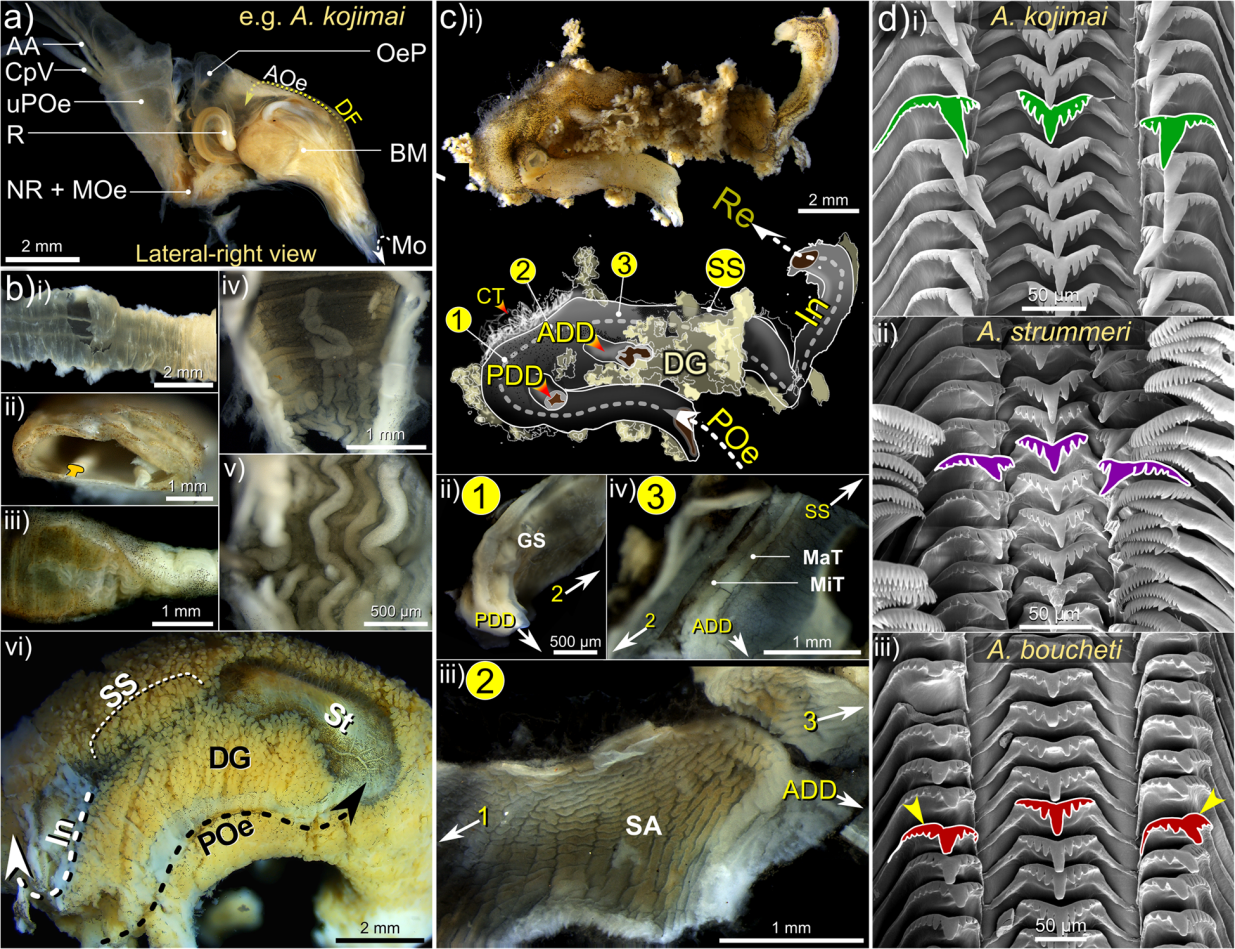
Elements of the alimentary and digestive system in *Alviniconcha.* Micrographs depict: **a** the anatomy of the buccal cavity; **b** internal and external morphology of the alimentary canal including: i) upper posterior oesophagus (uPOe) – posterior to the osophageal nerve ring encircling the mid-oesophagus (NR + MOe) – with ventral folds clearly visible and ii) transverse incision of uPOe immediately prior to mid-posterior oesophageal juction (in iii and iv) with T-profile of ventral folds highlighted. In iii) the mid-posterior oesophageal junction is marked by a decrease in diameter and (iv) the emergence of numerous dorsal folds, which rapidly become established (v). In vi) the lower posterior oesophagus (POe) enters the visceral haemocoel and communicates with the stomach posteriorly: arrow indicates the direction of travel. A schematic overview of stomach (St) anatomy in presented in **c** including micrographs of several anatomical features; in **d** scanning electron micrographs of pristine posterior regions of radulae from each species are provided. Pictures in **a**-**c** are from a formalin-fixed *A. kojimai* specimen. Additional abbreviations: AA Anterior aorta; ADD Anterior digestive duct; AOe Anterior oesophagus; BM Buccal mass; CpV Cephalopedal Vein; CT Connective tissue; DF Dorsal fold; DG Digestive gland; GS Gastric shield; In Intestine; MaT Major typhosole; MiT Minor typhosole; Mo Mouth; OeP Oesophageal pouches; PDD Posterior digestive duct; R Radula; Re Rectum; SA Sorting area; SS Style sac

The anterior-most cavity of the cephalopedal haemocoel (i.e. the buccal cavity) is relatively narrow, laterally, and tall, dorsoventrally (Figs. 4 and 5, Additional file 1 + model, Additional file 2 + model). In medial sagittal cross-section it appears roughly inverse-triangular, comprising; 1) the ventrally directed region that houses the circumoesophageal complex, comprising the oesophageal nerve ring and associated circulatory system branching out within the foot; 2) the anterior region – within the snout – housing the buccal complex (i.e. mouth, buccal mass, anterior oesophageal opening and anterior oesophagus, salivary glands and buccal ganglia) and nerves/blood-supply to the cephalic tentacles and; 3) the posteriorly directed (and dorsal-most) region, through which the upper posterior oesophagus (posterior to the oesophageal nerve ring), several nerves and two blood vessels all pass (Figs. 4 and 5, Additional file 1 + model). The muscle wall from the right-to-dorsal side of the head-foot is over twice as thick as the left, with the cephalopedal haemocoel displaced left of the medial line posterior to the buccal mass. Posterior to the buccal cavity, the cephalopedal haemocoel widens and flattens dorsoventrally, with irregular undulations visible externally on the pallial floor. When present, the anterior-most region of the gonad is visible as a bulge in the pallial floor on the far-left side of the pallial cavity level with the anterior end of columellar muscle and continues posteriorly, until it meets with the pallial skirt and thus the cardio-renal complex (Fig. 4, Additional file 1 + model, Additional file 2 + model).

The visceral hump (and the visceral haemocoel within), located posterior to the pallial skirt, occupies ~ 1 whorl, comprising the digestive glands, the cardio-renal complex, lower posterior oesophagus, stomach and intestine (Fig. 4, Additional file 1 + model and Fig. 5b). The short intestine soon merges into the rectum in the anterior-most region of the visceral hump, returning along the far-right side of the pallial cavity for much of its length.

#### Species-specific morphoanatomical characteristics

Muscle tissue in frozen specimens is pale pink in *A. boucheti* but decidedly darker in *A. kojimai* and *A. strummeri* (Fig. 3). When contracted, the snout appears to be wider distally in *A. kojimai*, at times almost pear-shaped; in *A. strummeri*, near-square shaped and narrower and in *A. boucheti,* the snout is very broad and round. However, these differences do not hold true for live specimens (F. Pradillon, unpublished observations). The mouth is encircled by bright-red tissue in frozen specimens of *A. kojimai* (Additional file 3v).

### Alimentary system overview

**Fig. 6:**
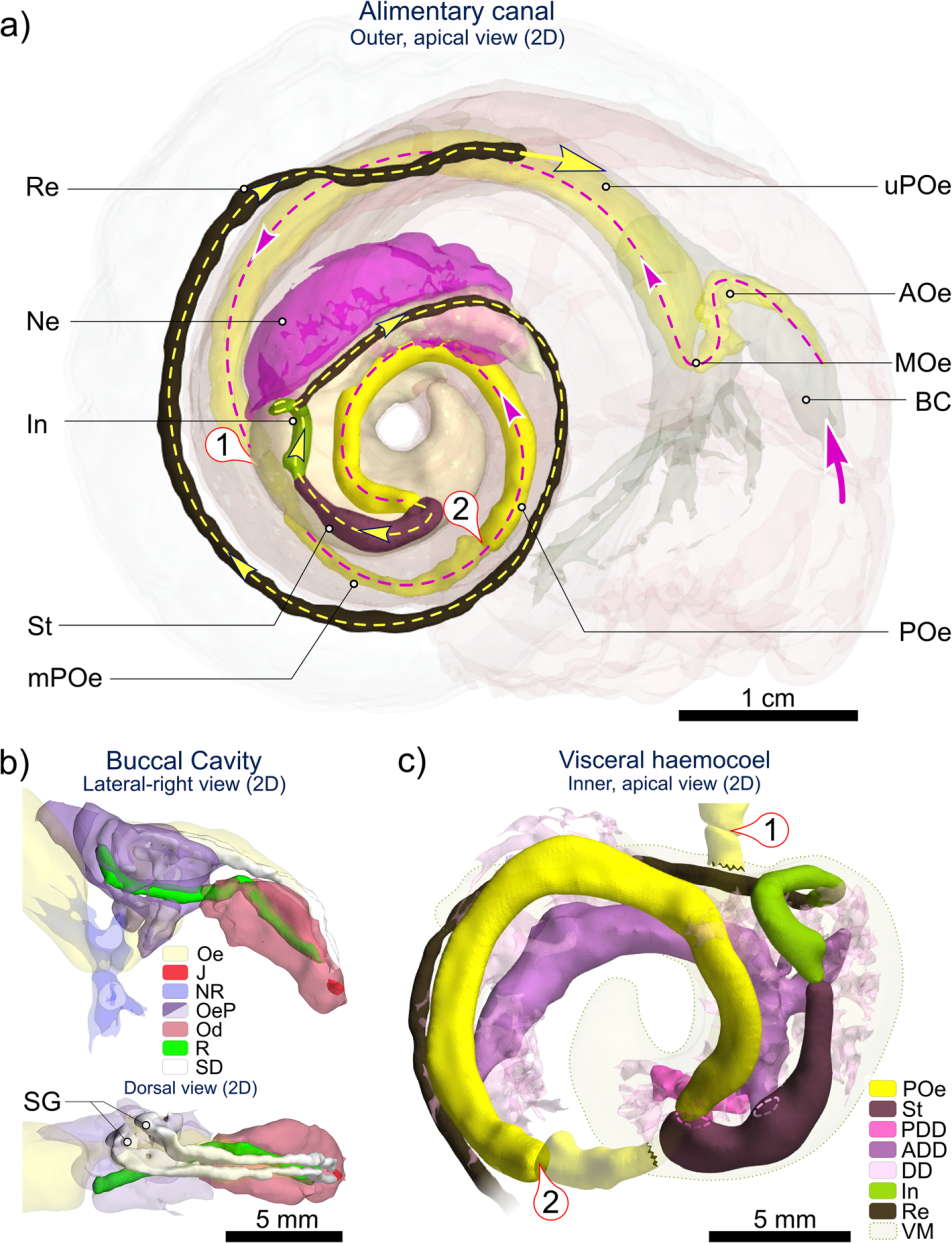
2D (flattened) visualisation of the *Alviniconcha* alimentary system based on CT. Images are from the CT-scanned *A. kojimai* specimen. Pictured: **a** overview of the alimentary system in *Alviniconcha* (excluding digestive glands), displaying the route of ingested material on its passage between the mouth (large pink arrow) and the end of the rectum (Re); **b** enlarged view of buccal cavity with jaws (J) and radula (R), nested in the curved odontophore (Od), with associated salivary ducts (SD) terminating in salivary glands (SG) left of the oesophagus (Oe) and blind oesophageal pouches (OeP); **c** enlarged inner-side view of visceral mass (VM) showing the relative positions of the stomach (St) and associated anterior and posterior digestive glands (ADD, PDD), lower posterior oesophagus (POe), intestine (In) and rectum. ① is upper-to-mid-posterior oesophageal junction; ② is mid-to-lower posterior oesophageal junction. Figure is of flattened 2D renderings with exact scales. Additional abbreviations: AOe Anterior oesophagus; BC Buccal cavity; DD digestive diverticula; MOe Mid-oesophagus; mPOe Mid-posterior oesophagus; Ne Nephridium; NR Oesophageal nerve ring; uPOe Upper posterior oesophagus

As in all gastropods, the alimentary system is U-shaped. In order of occurrence, the descending limb is composed of a mouth; buccal mass; relatively small salivary glands, and the long oesophagus, which extends from the buccal cavity to the visceral hump, where it turns abruptly to enter the returning stomach posteriorly (Figs. 4, 5, 6, Additional files 1 and 2 + models). Following conventional nomenclature, the oesophagus is composed of three regions delineated by the location of the oesophageal nerve ring; the anterior and posterior regions are anterior and posterior to the nerve ring, between which is the mid-oesophagus, the short, constricted region that passes through the oesophageal nerve ring. For additional clarity however, the long posterior oesophagus is subdivided again herein into three regions, distinguishable by changes in diameter and their internal morphology (Figs. 4, 5, 6, detailed later): the upper- (extending most of the cephalopedal haemocoel), mid- (posterior-most cephalopedal haemocoel) and lower-posterior oesophagus (almost entirely within visceral haemocoel). The returning limb of the ‘U’ comprises the small stomach, which tapers anteriorly to form the style sac, the associated digestive glands, and ultimately the morphologically similar short intestine and much longer rectum.

### Buccal complex

The buccal mass is small at 3–4 mm in length (SH ~ 40–60 mm) and heart-shaped, narrowing ventrally and anteriorly (Fig. 6). Salivary glands are found in close association with the oesophagus, dorso-anterior to the oesophageal nerve ring but posterior to the buccal mass (Figs. 4, 5, 6, Additional file 1 + model). They communicate with the buccal cavity via separate, narrow dorsal ducts, entering dorsally above the jaws (Fig. 6). Orientated as an inverted “V” on the dorsal buccal wall, the two simple jaws are light brown, semi-circular, fragile, with minutely denticulate cutting edges (Fig. 6, Additional file 6a). The radular sac (and posterior end of radula) lies to the right of the oesophagus and is slightly coiled, dorsally (Figs. 5a, 6, Additional files 1 and 2 + models).

### Radular morphology

#### General observations

In all cases, radulae are taenioglossate with a 2–1-R-1-2 tooth arrangement including a central wider-than-long, fairly solid rachidian tooth, flanked on either side by a row of lateral teeth and two rows of marginal teeth, that are longer-than-wide and more flexible (Fig. 5d). The rachidian base is twice as wide as its cusp. The rachidian cusp’s central denticle is broad, conical and flanked by 4–6 (usually 5) narrower lateral denticles on either side, decreasing progressively in length. The centre of the rachidian is inflexed, extending laterally to form a low transverse M-shaped supporting ridge, basal to the rachidian cusp. In the lateral teeth, the base is wider than the cusp with sides curled dorsally and a sub-cuspid inflection forms a broad, poorly defined, basal denticle. Each lateral tooth’s outer edge is greater than twice the length of inner edge, pushing lateral teeth towards the rachidian when the radular membrane lies flat. Lateral tooth cusps are similar in width and shape to the rachidian cusp, though the cusp profile is less angular. Each lateral tooth’s central denticle is tetrahedral, broader-based than rachidian’s central denticle and flanked by 3 inner and 4–5 outer denticles of much shorter length. The inner and outer marginal teeth are the longest teeth of the radula and very similar in appearance within and between species, with the width of the marginal-teeth cusps being about ~ 1.5 x that of the base. The marginal teeth are inflexed centrally forming a shallow, longitudinal, supporting ridge (wider and more pronounced distally) and are adorned with squared-off, rake-like cusps with numerous congruent denticles continuing for a short length marginally (numbering ~ 21–24). The outer edge of the outer marginal tooth is tightly curled along its length. No interspecific differences were identified in marginal-tooth morphology.

Radular teeth appear more open anteriorly where the radular membrane curves over the surface of the chitinous sheath that houses the odontophore cartilage. Teeth here are faintly yellow/brown as opposed to colourless (remaining posterior teeth) and notably eroded (Additional file 6b).

#### Species-specific radular characteristics

Radular morphology differs slightly in each species (Fig. 5d). Radula lengths (RL) depended on size but were similar in each species at a given SH, based on the limited number that were measured. In *A. kojimai*, SH of 50.4–78.8 mm equated to RL of 6.46–10.2 mm (*n* = 3), in *A. strummeri* SH of 46.1 and 51.6 mm equated to RL of 6.35 and 6.60 mm respectively and in *A. boucheti,* for SH of 52.0 and 66.6 mm, RL were 6.9 and 9.7 mm respectively. *Alviniconcha kojimai* possesses ~ 35–37 transverse rows of teeth mm^− 1^, *A*. *strummeri* ~ 43 rows mm^− 1^ and in *A*. *boucheti* ~ 32–35 transverse rows mm^− 1^. The aforementioned M-shaped supporting ridge of the rachidian is robust in *A. kojimai*, less so in the other species, Fig. 5d). The inner and outer-halves of lateral-tooth cusps appear near-symmetrical in *A*. *kojimai* and *A. strummeri*, however the outer half is weakly inflated in *A. boucheti* (Fig. 5d, Table 2). In *A*. *kojimai*, lateral-tooth central denticles are longer than rachidian central denticles, while in *A*. *strummeri* and *A*. *boucheti*, the lateral-tooth and rachidian central-denticle lengths are roughly congruent (Fig. 5d, Table 2).

### The oesophagus and proximal tissues

The anterior oesophagus, exiting the buccal mass, quickly becomes voluminous, with irregular, blind, dorsal pouches directed posteriorly (Fig. 5a). Two conspicuous parallel, dorsal longitudinal folds are visible internally through the delicate and translucent lining (Fig. 5b-i), originating from the roof of the buccal cavity (dorsal fold origin, ‘DF’, in Fig. 5a). These two folds are T-shaped in cross-section and ca. 1 mm apart, forming an alimentary channel (Fig. 5b-ii). The oesophagus then descends abruptly (ventrally) while spiralling dextrally, becoming the highly constricted mid-oesophagus as it passes through the narrow oesophageal nerve ring and turns towards the right with the alimentary channel now ventral. It then more than doubles in width laterally (Fig. 5a, b) forming the (upper) posterior oesophagus on its ascent towards the region of the cephalopedal haemocoel beneath the pallial floor. This upper region of the posterior oesophagus continues for ~ 0.75 whorls posteriorly with no change in morphology or diameter (as depicted in Fig. 5b). It is flanked on the right by the returning cephalopedal vein and (at least) the right visceral connective and on the left by the anterior aorta – delivering blood to the head-foot – originating from the ventricle (Fig. 4, Additional files 1 and 2 + models). This artery passes over the upper-posterior oesophagus dorsally from left-side-to-right, just prior to descending into the buccal cavity (~ 8–10 mm posterior to oesophageal nerve ring, Fig. 4, Additional files 1 and 2 + models).

**Fig. 7:**
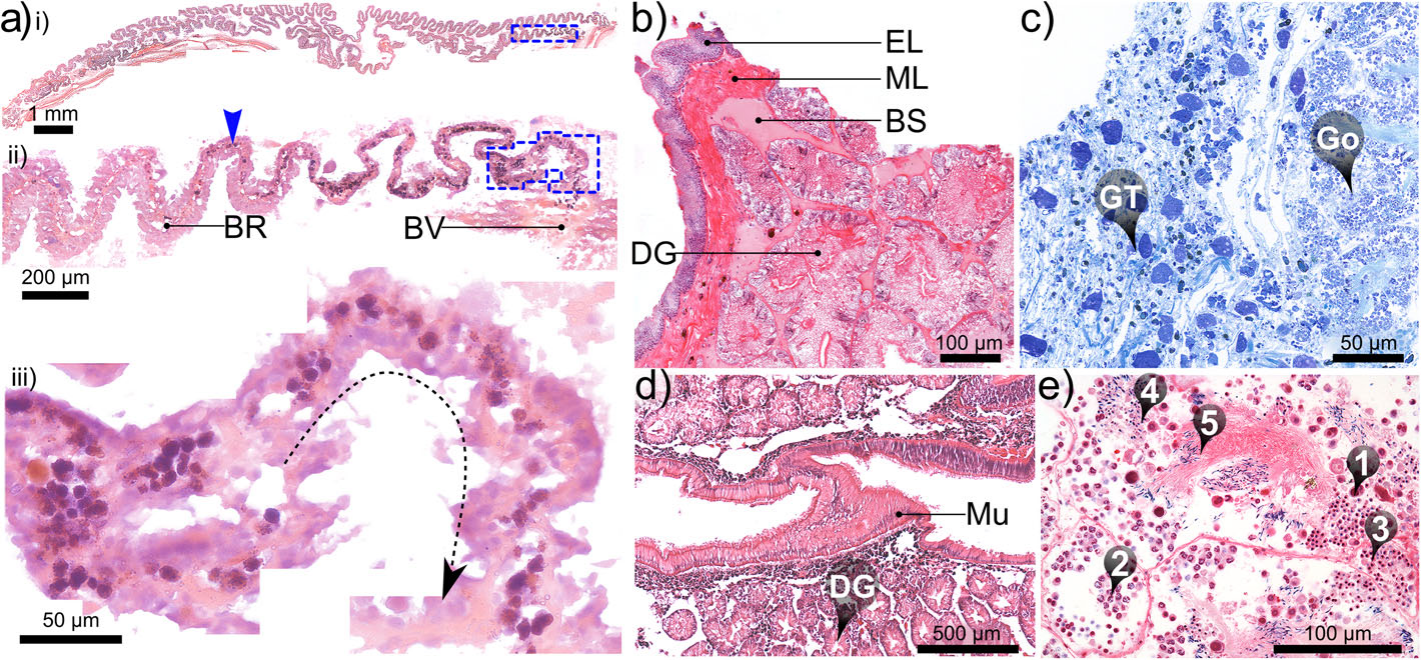
Histological micrographs of select tissues in *Alviniconcha.***a** Increasingly magnified views (corresponding to blue-boxed regions of previous image) of branchial filaments in oblique longitudinal section: arrowhead in ii) identifies the accumulation of cellular debris in the blood lacuna within the bacteriocyte region (BR) with iii) a higher magnification view of the junction with a large blood vessel (BV in ii, likely the secondary efferent branchial vessel); **b** transverse section through the outer layers of the posterior digestive gland (DG) bathed in blood sinuses (BS) and enclosed in delicate muscle and epithelial layers (ML and EL); **c** section cut from granular tissue (GT), a spongy tissue composed of a loose, fibrous matrix, densely populated with accretions of various sizes, found throughout the cephalopedal haemocoel but particularly around the mid-posterior oesophagus (not pictured), which passes through the gonad (Go), when present; **d** granular deposits also accumulate in connective tissue between the DD and the stomach mucosa (Mu); **e** various stages of spermatogenesis could be readily identified in males examined histologically: spermatogonia, spermatocytes, spermatids, spermatozoids and spermatozoa (❶ - ❺ respectively). Image mosaics **a** & **d** are from 7 μm-thick paraffin sections (formalin-fixed); **b**, **c** (both alcohol-fixed) & **e** (formalin-fixed) are from 2 μm-thick LR-white sections. All tissues are from *A. kojimai*, except (**c**), which is from *A. strummeri*

Much of the cephalopedal haemocoel is occupied by loosely bound, diffuse, almost spongy, granular connective tissue. This tissue is darker and so more conspicuous posteriorly (due to presence of fine black particulates, Fig. 7c); in larger specimens, the dark-grey to black colouration extends farther anteriorly. It occupies the lateral space in the cephalopedal haemocoel between the lighter-coloured anterior aorta, oesophagus and cephalopedal vein, which thus form three conspicuous, approximately parallel, lighter bands, discernible externally through the pallial floor. When present in adult specimens, the left-displaced gonad overlies the anterior aorta at this point, extending almost 0.5 whorls anteriorly from the posterior end of the cephalopedal haemocoel (Fig. 4, Additional files 1 and 2 + models). Shortly before the end of the cephalopedal haemocoel, the upper-posterior oesophagus rapidly decreases in diameter at the junction with the shorter mid-posterior oesophagus (Fig. 5b-iii); the latter is longitudinally compressed, weakly convoluted and mostly obscured by granular connective tissue (itself embedded in gonad where present, e.g. Fig. 7c). Over its short length (a few mm only), the mid-posterior oesophagus continues posteriorly but progressively to the right until it is flush with the cephalopedal vein, shortly after which it then deflects left (towards the cardiorenal complex) on its approach to the visceral haemocoel. Internally, the mid-posterior oesophagus is also characterised by multiple additional, transversely pleated folds, originating dorsally with the change in oesophageal diameter, counter-face to the existing ventral alimentary canal (Fig. 5b-iv, −v). Granular connective tissue in this region is tightly bound to the mid-posterior oesophagus and more spatially restricted, almost jet black in colour and vaguely glandular in appearance (tissue appears convoluted, but with no evident ducts or tubules in histological sections, Fig. 7c). The junction between the mid- and lower posterior oesophagus is demarcated by a doubling in diameter and changes in internal morphology, being no longer compressed, with only a single conspicuous ventral fold remaining alongside numerous, delicate, longitudinal ridges. As it enters the visceral haemocoel it becomes partially enveloped in the left, posterior digestive gland (Fig. 5b-vi). The lower posterior oesophagus continues along the abapical-to-adaxial (inner-left) side of the visceral hump passing the more abaxial stomach, after which it turns abruptly to enter the stomach posteriorly (Figs. 4, 5, 6, Additional files 1 and 2 + models). Both are partially visible at the surface amongst digestive diverticula at this point (Fig. 5-vi), beneath a layer of transparent epithelial tissue with a thin underlying layer of muscle (identified in histological sections, Fig. 7b). The single remaining internal oesophageal fold, which in the lower posterior oesophagus is only slightly convoluted, becomes enlarged and concertinaed, as it enters the stomach abaxially and extends anteriorly (and adapically) for about one fifth of the stomach interior.

### Gastrointestinal tract

**Fig. 8:**
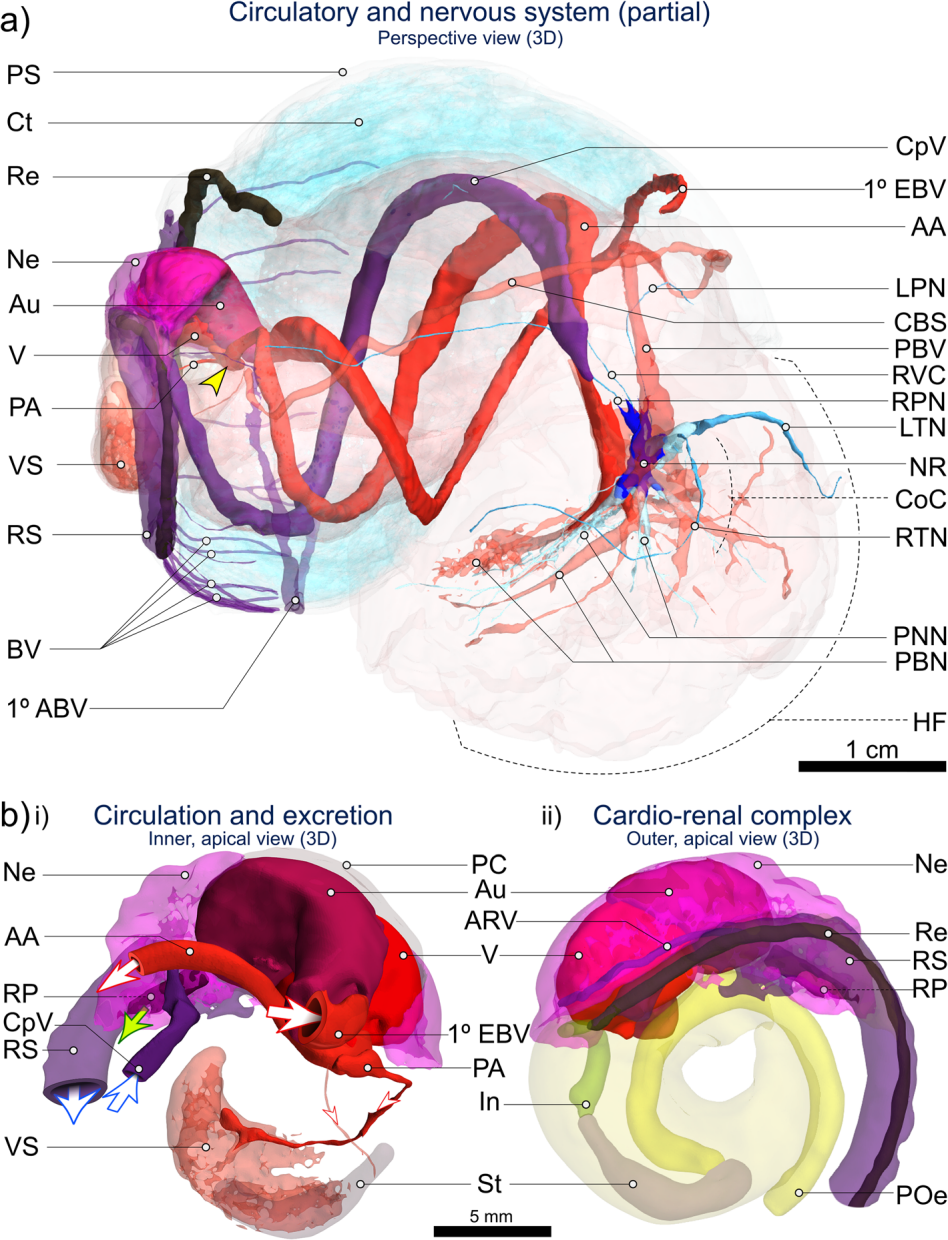
CT-based 3D visualisation of the circulatory, nervous and excretory systems in *Alviniconcha.* CT-scanned *A. kojimai* specimen: **a** 3D overview of cardio-renal complex, sinuses, vessels and nerves identified and visualised from CT volumes (N.B. pedal sinus not segmented, dorsal-most pallial vessels not shown, see Additional file 1); **b** Inner (i) and outer (ii) apical views of cardio-renal complex: i) Schematic derived from CT visualisation depicting the arrival of blood to the auricle (Au) via the primary efferent branchial vessel (1° EBV), after which blood exits the ventricle (V) via the larger anterior aorta (AA ~ > various cephalopedal sinuses) from which two smaller posterior vessels branch off, including the posterior aorta (PA ~ > visceral arterial sinus VS). Blood from rectal sinus (RS) later drains into the primary afferent branchial vessel – 1° ABV in a) – via lateral blood vessels (BV). Blood returning from head-foot to nephridium (Ne) arrives via the cephalopedal vein (CpV) and then passes to the afferent renal vein (ARV) visible in (ii) running along the right side of the nephridium, parallel with the start of the rectal sinus. Blood-vessel openings have been drawn by hand and are indicative only. Oxygenated blood represented by red vessels and arrows, deoxygenated blood by purple vessels and arrows. Oxygen content in rectal sinus likely decreases along its length. Green arrow in (i): excretion from the nephridium into the pallial cavity is via the renal pore (RP). Figure is of 3D renderings with exaggerated perspective, thus scales are approximate. Additional abbreviations: CBS Co-marginal blood sinus; CoC Circumoesophageal complex; Ct Ctenidium; HF Head-foot; In Intestine; LPN Left pallial nerve; LTN Left tentacular nerve; NR Oesophageal nerve ring; PBN Pedal blood network; PBV Pallial blood vessel; PNN Pedal neural network; POe Lower posterior oesophagus; PS Pallial skirt; Re Rectum; RPN Right pallial nerve; RTN Right tentacular nerve; RVC Right visceral connective; St Stomach

The stomach is very small in relation to animal size (< 0.05% of soft-tissue volume, Fig. 4, Additional files 1 and 2 + models) and is characterised anteriorly by a simple, linear style-sac but no crystalline style and a sorting area, with the gastric shield and digestive-gland ducts located more posteriorly (Fig. 5c). Much of the external stomach surface is obscured from view, being almost entirely enveloped in two digestive glands composed of furcate digestive diverticula (Fig. 5b-vi). Histological analyses revealed the presence of particulate material both between the digestive diverticula and in the layer of connective tissue found between diverticula and the stomach lining, similar in appearance to the granular connective tissue in the cephalopedal haemocoel (Fig. 7d). The boundaries of each digestive gland are poorly delineated but the digestive ducts – one for each gland – were readily identifiable in dissections (Fig. 5c-i) and during CT analyses (Figs. 4, 6, Additional files 1 and 2 + models). The opening of the adaxially orientated, posterior digestive duct is immediately anterior to that of the lower posterior oesophagus, both located in the posterior-most region of the stomach (Fig. 5c). The opening of the much-larger anterior digestive duct is located posterior to the style sac. It communicates with the stomach adaxially but then extends anteriorly, parallel to the descending lower posterior oesophagus along its adapical side (Figs. 4, 5c, Additional files 1 and 2 + models). Internally, the anterior digestive duct opens opposite to the sorting area (Fig. 5c-iii) and is demarcated by the beginning of the minor and major typhosoles (Fig. 5c-iv). The minor typhosole only extends half the length of the style sac but the major typhosole persists as a simple fold along the interior of the S-shaped intestinal tract with which the style sac communicates. In the current study, the end of this fold is considered the juncture between the intestine and the rectum, where the rectal sinus begins (Fig. 8c). The rectum and rectal sinus return anteriorly for most of the length of the animal enclosed in the mantle, which thickens to meet the pallial floor on the right (Fig. 4, Additional files 1 and 2 + models). Regions of moderate rectal distension are found intermittently along its length where contents are present inside, particularly towards the anus.

The foregut (anterior to the stomach) mainly contained mucus identifiable in preserved *A. kojimai* and *A. strummeri* specimens as fluffy, slightly compacted material, occasionally punctuated with particulate matter of various forms. Few breaks in these contents were found suggesting the intake of mucus had occurred prior to fixation, perhaps as a result of handling stress. Similar but patchy contents were also found in the stomach, however, the style-sac region and the hindgut beyond were characterised by contents punctuated with crystalline debris, particularly evident in CT volume data (voxels with greyscale levels comparable to calcareous shell, Additional file 4). In fact, the presence of this crystalline material made it relatively easy to follow the rectum’s progress anteriorly in CT volume data.

### Circulatory, excretory and nervous system

**Fig. 9:**
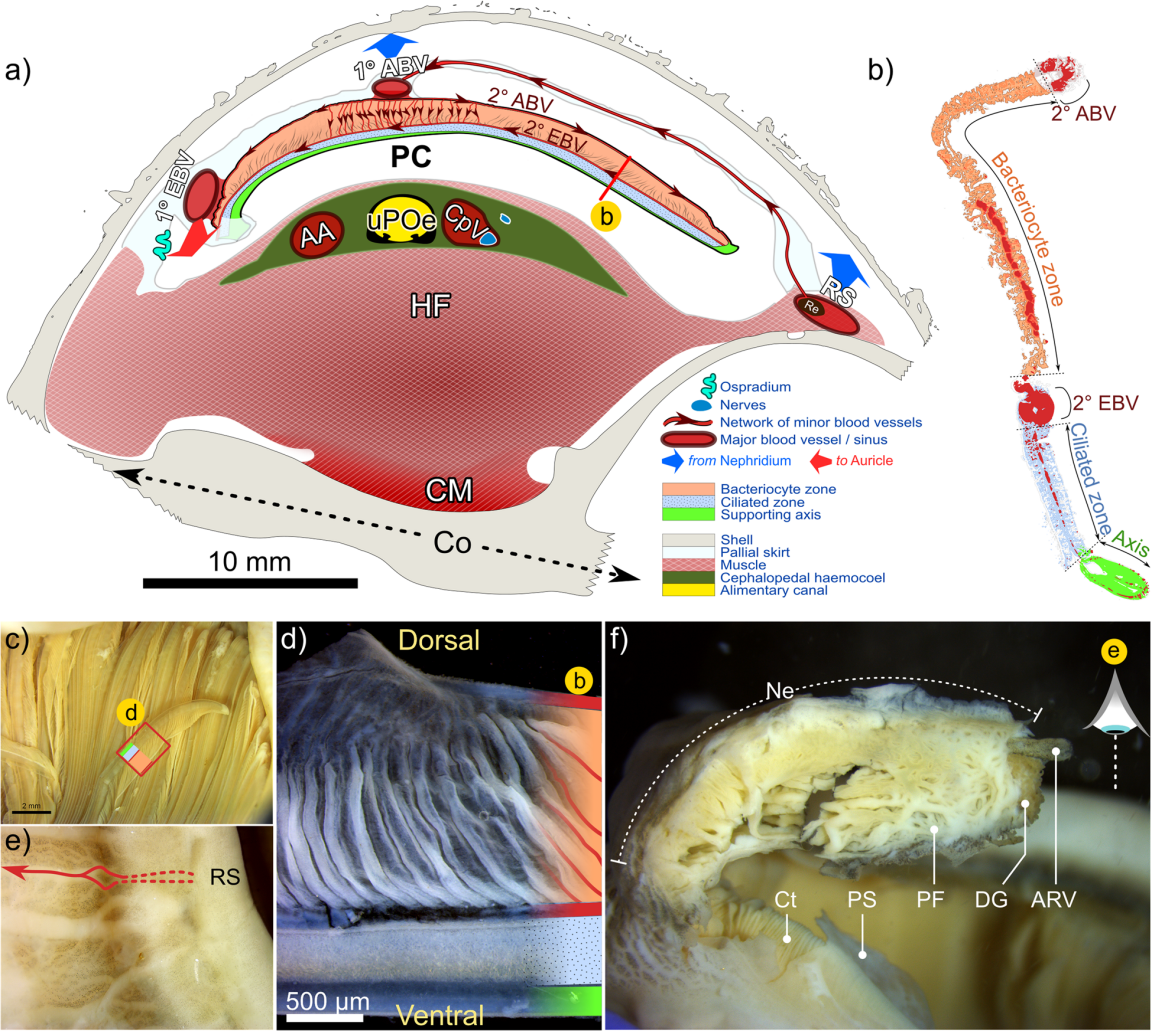
Summary of branchial anatomy and proximal tissues. Schematic in **a** depicts arrangement of gill filaments in relation to overall transverse cross-sectional anatomy at pallial cavity’s widest point (~ 0.25 whorls behind buccal cavity). Blood circulation is based on CT data, whole-filament histological staining (sections and unembedded) and details recorded during dissection. Though not depicted, areas in cephalopedal haemocoel not occupided by blood vessels or the alimnetary canal, were filled with spongy connective tissue full of particulate deposits. Depicted in **b** a branchial filament in sagittal section approximately located along the red line marked on the filament in **a**. The colour coding used in (**a**) and (**b**) is repeated in (**c**) and (**d**) for comparative purposes. Ctendium pictured in **c** is viewed ventrally: with most of filaments extending dorsally from the ventral support axes. One filament however has been displaced sideways and has been colour coded according to its abnormal orientation. Close up, these filaments appear as depicted in **d**. The micorgraph in **e** is of the dorsal face of the mantle on the far-right side of the animal, along which the the rectal sinus (RS) runs. Arrows indicate the blood vessels that deliver blood to the primary afferent branchial vessel (1° ABV) of the ctenidium from the rectal sinus. Image **f** is of the nephridium (Ne, with a transverse cut), which abuts directly with the posterior-most branchial filaments (Ct). Primary folds (PF) and the afferent renal vein (ARV) can be seen, along with traces of the digestive gland (DG). Additional abbreviations: 2° ABV secondary afferent branchial vessel; 1° / 2° EBV primary / secondary efferent branchial vessel; AA Anterior aorta; AOe Anterior oesophagus; CM Columellar muscle; Co Columella; CpV Cephalopedal Vein; CM columellar muscle; HF Head-foot; PC Pallial cavity; PF Pallial fringe; PS Pallial skirt; RS Rectal sinus

The heart’s single ventricle and auricle are relatively small, together occupying around 0.5% of the soft-tissue volume in *A. kojimai* and in *A. strummeri* (derived from CT segment volumes of the heart and the total soft tissue). Oxygenated blood from the lacunae of branchial filaments appears to drain into the primary efferent branchial blood vessel (Fig. 9a), which widens considerably as it travels the length of the ctenidium posteriorly on its left side – parallel to the gill axis – until finally, it makes an abrupt turn dorsally to enter the auricle (Fig. 8b, note that blood-vessel openings in this figure are hand-drawn and indicative only). Blood flows from the reservoir in the auricle into the muscularised ventricle chamber (Additional file 5c), where the contraction of a dense matrix of muscle fibres – evident in histological section (Additional file 5d) – drives the blood via the large anterior aorta towards the head-foot, and via the much smaller posterior aorta towards the visceral sinus (Fig. 8, Additional file 5b, but also Additional files 1 and 2 + models). In the buccal cavity, the anterior aorta branches into a blood network that radiates from the oesophageal nerve ring towards: the arterial pedal sinus (ventrally); the buccal mass (anteriorly); the cephalic tentacles (laterally) and – via a vessel that doubles back dorso-posteriorly – towards the left side of the pallial margin to supply the co-marginal blood sinus (Fig. 8a). The posterior aorta supplies blood to the arterial visceral sinus (Fig. 8b), which extends as several branching channels among the digestive diverticula visible on the right side of the visceral hump (Additional file 5b). Blood returns to the nephridium from the head-foot via the prominent cephalopedal vein (Fig. 8b) and presumably, from the venous visceral sinus via a visceral vein, however the latter was not apparent during dissections could not be readily identified in CT scans.

The nephridium forms a cradle around the right half of the pericardial cavity, slightly invading the pallial skirt anteriorly, abutting the most posterior branchial filaments (Fig. 8, Additional files 1 and 2 plus models, Additional file 5c). It is composed of a dense arrangement of primary and secondary folds, the former evident in cross-section (Fig. 9f). The cephalopedal vein communicates with the nephridium’s anterior wall and appears to merge with the afferent renal vein, which runs the length of the nephridium’s right wall (Fig. 8b and Additional-file-1 model), parallel to the nephridial gland, with a blind terminus in the posterior-most region of the nephridium. The renal pore, from which waste compounds from the nephridium are normally excreted (Fig. 8b, green arrow), is located near to the origin of the afferent renal vein, visible as a compressed slit in the right, posterior-most region of the pallial cavity. The rectal sinus bathes the rectum in haemolymph for almost its entire length, sending out dorso-lateral pallial blood vessels leftwards towards the primary afferent branchial vessel located medially in the pallial skirt (Figs. 8a, 9a, e and Additional-file-1 model), where haemolymph is then thought to drain into individual branchial filaments to be re-oxygenated (Fig. 9a).

The nervous system and neuronal characters were not examined by histology but observations during dissections and visualisations based on CT analyses suggest it is epiathroid, as described previously in *Alviniconcha* [3]. The presumptive arrangements of the pleural, cerebral and pedal ganglia (informed in part by previous studies) are indicated in the model in Additional file 1. However, further analyses using histology are necessary to establish whether these are true ganglia, i.e. paired neuron-cell cortices with centrically directed neurites (neurophil) and peripheral neuronal somata, connected to one another by neurite bundles (e.g. connectives, commissures). Dense nerve bundles were observed to extend ventrally from the oesophageal nerve ring into the foot’s metapodium, mesopodium and anterior-most propodium, based on dissections and CT visualisations (Fig. 8a). Three closely packed nerves extend anteriorly from each side of the oesophageal nerve ring and innervate four points on the buccal mass, two posterio-dorsally and two anterio-ventrally, as well as the two cephalic tentacles (Fig. 8a). The dorsal, posterior-most region of the oesophageal nerve ring appears disproportionately long on its left side. Several nerves also extend out more posteriorly (minimum of two from right side, four from left), of which the right and left pallial nerves (tracked towards mantle, Fig. 8a) and the right visceral connective could be confidently identified (the last, progressing along the cephalopedal vein, Fig. 8a).

### Ctenidium and associated tissues

#### General observations

The ctenidium – or gill – is hypertrophied and represents about 60% of the total, uncoiled body length (based on dissections) and a little over 10% of the animal’s total volume (based on CT data). The ctenidium extends the full length of the pallial cavity (e.g. Fig. 2b, Additional file 3b), fused with the pallial margin anteriorly and attached to the far left of the pallial roof along its axis. Its widens for 0.5 whorls and then tapers posteriorly, terminating just prior to the pallial skirt, at which point the primary efferent branchial vessel enters the pericardial cavity and the auricle of the heart (Fig. 8b, Additional files 1 and 2 + models). The osphradium, an olfactory sensory organ, extends from the pallial margin’s inhalant left side for about half the pallial cavity, approximately parallel to, and left of the ctenidium’s axis (Fig. 9a, and Additional files 2 + model and Additional file 5e). It takes the form of a central flange bordered by two much-less pronounced ridges. Tight ranks of branchial filaments extend laterally from the ctenidium’s axis. Most filaments terminate right of the pallial cavity’s midline; at their longest – above the anterior-most dorsal hump of the cephalopedal haemocoel – they occupy around 80% of the cavity’s width (Fig. 4, Additional files 1 and 2 + models). Filaments are adhered to the pallial epithelium for their proximal half, free-floating distally, where the afferent branchial vessel demarcates the right, medio-dorsal boundary of this adhered region (Figs. 4, 9a, c, Additional file 1 + model, Additional file 5f). Each filament is comprised of a rigid supporting rod along its ventral edge, a narrow ciliated zone and a much wider membranous region, which is delicate, plicate and highly vascularised (Fig. 9b, c, d). It is in this latter region that bacteriocytes are found, housing the bacterial symbionts (Fig. 9b). The primary afferent branchial vessel delivers blood to secondary afferent blood vessels, which run along the dorsal edge of each filament, while secondary efferent blood vessels lie along the boundary between the ciliated and bacteriocyte zones (Fig. 9a, d). Thus, the secondary branchial vessels frame the bacteriocyte region and are visibly linked by a vertical network of delicate, dorsoventral blood vessels (Fig. 9a, d). Histological examination of these blood vessels reveals the coagulation and transport of lysed biological material of unknown origin from the bacteriocyte region to secondary efferent blood vessels (Fig. 7a), providing putative evidence for bacterial assimilation.

#### Species-specific characteristics

Filaments in *A. strummeri*, and particularly *A. kojimai*, are wide dorsoventrally (Additional file 5d); the dorsal half of each are creamy white in appearance and engorged (partially visible in Additional file 5b). In *A. boucheti*, filaments are notably darker (lacking engorged tissue, Fig. 2b), more mucous and narrower dorsoventrally (Additional file 5d).

### Reproductive system

Gonad size varied – in terms of its extent anterio-posteriorly – from being restricted to the posterior region of the cephalopedal haemocoel, to extending as far as the anterior end of the columellar muscle. Observations during dissections gave the impression that this variability was not a function of size, however the number of specimens dissected was relatively low and gonad size was not quantitatively assessed. The smallest individual dissected however, an *A. kojimai* specimen with SH 37.8 mm, had no visible gonad. Limited data available from the current study indicate that these *Alviniconcha* spp. are gonochoric. In those specimens that were reliably sexed, SH for each sex were as follows: *A. kojimai* ♀ 41.5–78.8 mm (*n* = 8), ♂ 52.6–77.4 mm (*n* = 5); *A. strummeri* ♀ 53.7 mm, ♂ 46.1–51.6 mm (*n* = 4) and; *A. boucheti* ♀ 65.9–77.6 mm [*n* = 3], ♂ 59.8 mm. In frozen individuals, gonads confirmed by tissue smear are creamy white and opaque in females, while gonads in males are brown-to-orange and some internal detail is visible through the epithelium. Mean defrosted oocyte diameters were small at 50.69 μm ± 4.5, measured in *A. kojimai* only (three individuals for which eggs were intact, minimum of five replicates per individual). However, the meiotic status of these oocytes was not confirmed. Detailed analyses of the female reproductive system were confounded by a lack of chemically fixed female specimens in the current study. Consequently, the presence of a seminal receptacle as described in [3], was not confirmed in the frozen females available. Chemically fixed male gonads were opaque and mainly composed of spongy tissue, with a network of delicate tubules that met posteriorly to form a compact array of seemingly blind, branching ducts. Internal gonad structure was poorly resolved in CT data but when examined histologically (*A. kojimai* and *A. strummeri* only), spongy tissue was characterised by all stages of spermatogenesis from spermatogonia to spermatozoa (Fig. 7e) and the terminal branching ducts were replete with mature spermatozoa (not shown). A simple, putative gonoduct in the form of continuous tightly compressed flap and running parallel with and ventral to the rectal sinus, was identified in fixed (male) specimens and in CT data (sex not verified) (Fig. 4a, Additional file 1 + model, N.B. gonoduct is not complete). Based on these observations, it was not clear where this duct met with the gonad.

### Summary of differences between species

The morphological and anatomical differences between *Alviniconcha kojimai*, *A. strummeri* and *A. boucheti* are summarised in Table 2. Of these, interspecific variability in periostracal bristle lengths and arrangement have been validated in 90 specimens. Additional observations on soft-body morphology and internal anatomy are derived from a subsample of these specimens (see table legend). Together, these have been incorporated into amended species’ diagnoses, below.

### Taxonomy

Phylum MOLLUSCA

Class GASTROPODA

Clade (Subclass, Order unassigned) CAENOGASTROPODA Cox, 1960

Superfamily ABYSSOCHRYSOIDEA Tomlin, 1927

Family PROVANNIDAE Warén & Ponder, 1991

### Genus *Alviniconcha* Okutani and Ohta, 1988

#### Type species

*Alviniconcha hessleri* Okutani and Ohta, 1988, by original designation, from hydrothermal vents in the back-arc basin of the Mariana Trough.

#### Remarks

The type species is *Alviniconcha hessleri* and the genus description is modified herein to include some variations between species. *Alviniconcha hessleri* was redescribed in [3] but the additional material examined was from localities where three *Alviniconcha* species occur (based on subsequent molecular analyses), none of them being *A. hessleri* [1].

#### Amended diagnosis

Shell dextral, globose, flexible (except columella), ornamented with regularly spirally arranged periostracal bristles. Bristles either of similar length (e.g. *A. strummeri*, and possibly *A. adamantis*, based on the appearance of the shell featured in Figure 2.6 of the Johnson et al. 2015 study [1]) or forming a repeating pattern of different lengths axially (e.g. *A. kojimai* and *A. boucheti,* and possibly *A. marisindica* and *A. hessleri*, based on the appearance of shells featured in Figures 2.1 and 2.4 of the Johnson et al. 2015 study [1]). Columellar surface either smooth (e.g. *A. boucheti*) or with a single fold (e.g. *A. kojimai* and *A. boucheti*). Operculum horny and ovo-quadrate. Foot demarcated either side by a lateral epipodial fold. Right flank possesses a descending neck furrow. Pallial margin adorned with stout, tapering papillae but no pallial tentacle. Rachidian with a distinct central cusp and several lateral cusps; lateral slender with a large cusp of variable length depending on the species and several small cusps; two marginals, triangular, with squared-off, rake-like cusps of 20–30 denticles.

### *Alviniconcha kojimai* Johnson, Warén, Tunnicliffe, Van Dover, Wheat, Schultz & Vrijenhoek 2015

#### Type material

SMNH type collection 8577, preserved in 95% ethanol. GenBank # KF467685.

#### Type locality

Tow Cam vent site, 20°19.0760S, 176° 8.2580 W, 2714 m depth in the Lau basin. ROV Jason II, dive 142.

#### Remarks

Original designation based on molecular diagnoses only. Current study describes associated anatomy based on dissections, histology, scanning electron microscopy (SEM) and the examination and 3D segmentation of CT data. These data suggest that specimens of *Alviniconcha kojimai* inadvertently informed the re-description of the type species *A. hessleri* by Warén and Bouchet [3], based on specimen origins [1, 4] and identifiable periostracal-bristle arrangements of specimens featured in photographic plates from that study.

At the time of writing and based on current distribution data, the IUCN lists this species as endangered [34].

#### Material examined

Specimens collected from the Fatu Kapa vent field (within EEZ of Wallis and Futuna Islands) during the Futuna 3 cruise with the human-operated vehicle, HOV, *Nautile*. When measured (*n* = 43), SH ranged from 37.8–89.1 mm. Periostracal-bristle arrangement examined in 61 frozen, 1 ethanol-, 3 formalin-fixed specimens. Dissections performed on 12 frozen, 1 ethanol-, 2 formalin-fixed specimens. Histology carried out on multiple tissues from 5 specimens. SEM analyses of radulae extracted from three frozen specimens. CT analyses were on the formalin-fixed voucher specimen with code MNHN-IM-2014-7972 (DNA extraction unsuccessful), which has been deposited at the Muséum National d’Histoire Naturelle, Paris (SH 72.3 mm). This specimen was collected live from the Aster’x vent site by HOV *Nautile* during dive # PL18–1839 of the Futuna 3 cruise (14°45′ 6“S, 177° 9’ 8”W, 1571-m depth on June 14th, 2012), fixed in 4% formalin, stored in 96% ethanol at room temperature (~ 5 yrs), then contrast-stained in 1%-Lugol’s iodine (I_2_/KI) and following CT scanning, returned to 96% ethanol at room temperature for storage.

#### Amended diagnosis

In addition to this species’ molecular diagnosis, several distinctive morphoanatomical characters have since been observed. Two distinct lengths of periostracal bristle. On last shell whorl, spiral rows of 1° bristles are ~ 5-mm long, separated by three (occasionally four) 0.5-mm long 2° bristles, with repeating axial pattern of 1° - 2° - 2° - 2° (− 2°) - 1°. Periostracum lifted in minute ridges between bristle bases both spirally and axially. Columella possesses columellar fold, displaced adapically within whorls, spiralling from apical whorl to 0.5 whorls before aperture opening. Head-foot is pink when alive (blue-purple where blood sinuses lie beneath); tentacles bright red in frozen specimens. Branchial filaments are broad dorsoventrally (up to 4 mm), where white-to-cream hypertrophied bacteriocyte regions give ctenidium a stripy appearance (thought to relate to elemental sulphur deposits [11]). Radula: cusp of rachidial tooth is notably inflexed (v-shaped); non-medial denticles of lateral-teeth cusps are approx. Uniform in length, and the central denticle is longer than that of the rachidial tooth.

#### Distribution

In addition to the reported distribution [1, 4], the species has been collected at hydrothermal-vent sites Stéphanie, Aster’x, Obel’x and Fati Ufu, located in the Fatu Kapa vent field near the Wallis and Futuna Islands.

### *Alviniconcha strummeri* Johnson, Warén, Tunnicliffe, Van Dover, Wheat, Schultz & Vrijenhoek 2015

#### Type material

SMNH type coll. 8573. Fixed in 4% seawater-buffered formaldehyde, stored in 80% ethanol. GenBank # KJ027398.

#### Type locality

Tui Malila vent site, 21° 59.4310S, 176° 34.1460 W, 1845 m depth in the Lau Basin. ROV Jason II, dive J2144.

#### Remarks

Original designation based on molecular diagnoses only. Current study describes associated anatomy based on dissections, histology and the examination and 3D segmentation of CT data. Although specimens of this species may have informed the re-description by Warén and Bouchet [3] of the type species *A. hessleri*, no evidence from re-examination of photographic plates in that study could confirm this. At the time of writing and based on current distribution data, the IUCN lists this species as vulnerable [35].

#### Material examined

Specimens collected from the Fatu Kapa vent field (within EEZ of Wallis and Futuna Islands) during the Futuna 3 cruise with the HOV *Nautile*. When measured (*n* = 3), SH ranged from 46.1–69.9 mm (*A. strummeri* specimens were smallest of three SW-pacific species, occurring in much lower numbers). Periostracal-bristle arrangement examined in 7 specimens (two in ethanol, five frozen). Dissections performed on 6 specimens (1 in ethanol, 5 frozen). Of these, histology carried out on multiple tissues from one specimen. SEM analyses of radulae extracted from two frozen specimens. CT analyses were on remaining ethanol-fixed voucher specimen with code MNHN-IM-2014-7973 and GenBank# MT010489, which has been deposited at the Muséum National d’Histoire Naturelle, Paris (SH 69.9 mm). This specimen was collected live from the Aster’x vent site by HOV *Nautile* during dive # PL18–1839 of the Futuna 3 cruise (14°45′ 6“S, 177° 9’ 8”W, 1571-m depth on June 14th, 2012), fixed and stored in 96% ethanol at room temperature (~ 5 yrs), then contrast-stained in 1%-Lugol’s iodine (I_2_/KI) and following CT scanning, returned to 96% ethanol at room temperature for storage.

#### Amended diagnosis

In addition to this species established molecular diagnosis, several distinctive morphoanatomical characters have since been observed. All periostracal bristles adorning last whorl are approximately congruent in length, axially and spirally. Bristles are long (already ~ 7 mm at SH ≤ 50 mm). No obvious inter-bristle ridges. Robust columella possesses pronounced columellar fold, displaced adapically within whorls, spiralling from apical whorl to 0.5 whorls before aperture opening. Head-foot is pink when alive (blue-pink where blood sinuses lie beneath); tentacles red-to-orange in frozen specimens. Branchial filaments are moderately broad dorsoventrally (up to 3 mm), where white-to-cream hypertrophied bacteriocyte regions give ctenidium a stripy appearance (thought to relate to elemental sulphur deposits [11]). Radula: cusp of rachidial tooth is notably inflexed (v-shaped); non-medial denticles of lateral-teeth cusps decrease progressively in size from central denticle; lateral and rachidial central denticles similar in length.

#### Distribution

In addition to the reported distribution [1, 4], the species has been collected at hydrothermal-vent sites Stéphanie, Aster’x, and Obel’x, located in the Fatu Kapa vent field near the Wallis and Futuna Islands.

### *Alviniconcha boucheti* Johnson, Warén, Tunnicliffe, Van Dover, Wheat, Schultz & Vrijenhoek 2015

#### Type material

SMNH type coll. 8575, Fixed in 4% seawater-buffered formaldehyde, stored in 80% ethanol. GenBank # KF467804.

#### Type locality

Mussel Hill vent site, 16° 59.410S, 173° 54.970E, 1973 m depth in the North Fiji Basin. ROV Jason II, dive J2152.

#### Remarks

Original designation based on molecular diagnoses only. Current study describes associated anatomy based on dissections, histology and the examination (only) of reconstructed CT data. These data suggest that *Alviniconcha boucheti s*pecimens inadvertently informed the re-description of the type species *A. hessleri* by Warén and Bouchet [3], based on specimen origins [1, 4] and identifiable periostracal bristle arrangements of specimens featured in photographic plates from that study. At the time of writing and based on current distribution data, the IUCN lists this species as endangered [36].

#### Material examined

Specimens originate from the Fatu Kapa vent field found within the French EEZ of the Wallis and Futuna Islands during the Futuna 3 cruise with HOV *Nautile*. For those measured (*n* = 13), SH in current study ranged from 49.4–77.6 mm. All specimens were frozen (branchial filaments from a subset of specimens were fixed for microscopic analyses). Periostracal-bristle arrangement was examined in 32 specimens. Detailed dissections performed on 4 frozen specimens. Histology carried out on formalin-fixed gill tissue from 2 specimens. SEM analyses of radulae extracted from three frozen specimens. CT analyses were on frozen voucher specimen post-fixed in 4% formalin with code MNHN-IM-2014-7974 and GenBank# MT010510, which has been deposited at the Muséum National d’Histoire Naturelle, Paris (SH 71.7 mm, CT data was not segmented). This specimen was collected live from the Fati Ufu vent site by HOV *Nautile* during dive # PL21–1842 of the Futuna 3 cruise (14° 45′ 36“S, 177° 11’ 6”W, 1530-m depth on June 16th, 2012), transferred to − 80 °C on board (stored for ~ 5 yrs), post-fixed in 96% ethanol at 4 °C, stored at room temperature, then contrast-stained in 1%-Lugol’s iodine (I_2_/KI) and following CT scanning, returned to 96% ethanol at room temperature for storage.

#### Amended diagnosis

In addition to this species established molecular diagnosis, several distinctive morphoanatomical characters have since been observed. Three distinct lengths of periostracal bristle. On last shell whorl, spiral rows of 1° ~ 5-mm long bristles are interspersed with a 2-mm long 2° bristle and two 0.5-mm long 3° bristles, with repeating axial pattern of 1° - 3° - 2° - 3° - 1°. No obvious inter-bristle ridges. Columella lacks columellar fold. Head-foot (including tentacles) is pale pink when alive and frozen (blue-grey where blood sinuses lie beneath). Branchial filaments are narrow dorsoventrally (up to ~ 2 mm), with brown, highly mucous bacteriocyte regions, giving the ctenidium a uniform brown-to-grey appearance. Radula: cusp of rachidial tooth only slightly inflexed; non-medial denticles of lateral-teeth cusps decrease progressively in size from central denticle; lateral and rachidial central denticles similar in length, but outer half of lateral teeth have an asymmetric inflated profile.

#### Distribution

In addition to the reported distribution [1, 4], the species has been collected at the hydrothermal-vent site Fati Ufu, located in the Fatu Kapa vent field near the Wallis and Futuna Islands.

## Discussion

The ability to discriminate species plays a critical role in the proposition and validation of ecological hypotheses. When this is not possible, it places constraints on the interpretation of data (e.g. being forced to pool *Alviniconcha* species when assessing chemical-substrate use during symbiont chemoautotrophy, [13]). Using 3D imagery in concert with detailed dissections and histological tissue preparations, the current study addresses this problem in three SW Pacific *Alviniconcha* species. The results reveal that SW Pacific species of *Alviniconcha* are not cryptic as has been previously suggested. All three species are readily identifiable based on periostracal-bristle characteristics and columellar morphology alone but in cases of ambiguity between *A. kojimai* and *A*. *boucheti*, visual appearance of the ctenidium, head-foot coloration, the presence of minute periostracal ridges in *A. kojimai* and species-specific radular features provide additional, distinguishing characteristics (summarised in Table 2).

Prior to the current study, a detailed record of the anatomy pertaining to *Alviniconcha* was restricted to the type species *A. hessleri* only ([2], with a revised description in [3]). The hypothesis that *Alviniconcha* spp. are cryptic likely stems from this early re-description [3], based on morphoanatomical observations from *Alviniconcha* specimens sampled at White Lady vent field in the North-Fiji Basin and Vai Lili and Hine Hina vent fields in the Lau Basin. In it, Warén and Bouchet report marked “individual variability” in *Alviniconcha* shell ornamentation and shape in both juvenile and adult specimens, ascribed to intraspecific phenotypic plasticity. However, even as this emended diagnosis of *A. hessleri* and the thus genus was being published in 1993, a second study emerged using specimens from the same sample locations, which identified high genetic diversity within the genus, indicating the presence of multiple species. At that time, it was not clear whether *A. hessleri* was even one of the species present at these sites; tissue from the type specimens was not available for DNA extraction. Studies over the course of the 20 years that followed have established the existence of six evolutionary lineages in *Alviniconcha* [1, 5–7] and consequently, five species in addition to *A. hessleri* [1]. We have learnt that five of these species occur in the Pacific and two of them, *A. hessleri* and *A. adamantis*, appear to be geographically isolated in the Mariana Back-arc Basin and Mariana Volcanic Arc respectively. Specimens from the Manus Basin, North-Fiji Basin and the Lau Basin in the SW Pacific are represented by subsets of the three species in the current study, *A. kojimai*, *A. boucheti* and *A. strummeri*. Taking these species distributions into account, it is very likely the *Alviniconcha* specimens examined by Warén and Bouchet [3] collected from the North Fiji and Lau basin were not *A. hessleri* and potentially not even from a single species. We have re-examined shell photographs of two of the adult specimens featured in that study from the Vai lili vent field in the Lau Basin, in so far as the resolution allows. The periostracal bristle arrangement of the first specimen in that study (i.e. Figure 45A, C in [3]) appears to follow the 1°- 2°- 2°- 2°- 1° pattern described for *A. kojimai* in the current study, with a few instances of the rarer variant where central 2° bristles are slightly longer than the adjacent 2° bristles. This latter arrangement superficially resembles that of *A. boucheti*, except the lattice-like appearance of the periostracal ridges visible in the first specimen photos, characteristic of *A. kojimai* only*.* The second specimen in the Warén and Bouchet 1993 study (i.e. Figure 45B in [3]) is almost certainly *A. boucheti*, on account of its very large size and distinct 1°- 3°- 2°- 3°- 1° bristle arrangement (the 2° being about half the length of the 1°). There is also extensive bristle loss and peeling of top periostracal layers in the pictured specimen and with it, the exposure of large areas of darker periostracum. While bare periostracum was quite common in all larger specimens, peeling was a feature that was only in the largest *A. boucheti* specimens (~SH 70+ mm) from the Wallis and Futuna volcanic region. Similar bristle loss and peeling is evident in the very large specimen of *A. boucheti* pictured in the Johnson et al. 2015 study (i.e. Figure 2.3 in [1]), suggesting this is a common feature in larger *A. boucheti,* perhaps symptomatic of prolonged exposure to corrosive site conditions. Our shell-based species assignments for *Alviniconcha* specimens pictured in [3] are in agreement with those from Denis et al. [4], who identified the presence of multiple SW Pacific *Alviniconcha* species – since confirmed as *A. kojimai* and *A. boucheti* [1] – also from Vai lili and White Lady (North-Fiji basin). Photos of juvenile and post-larval *Alviniconcha* are also featured in Warén and Bouchet [3], from the Vai lili and White Lady vent fields respectively, with descriptions based on samples from both the Lau and North-Fiji Basin. The juvenile photos displayed are of two distinct morphotypes. Given what we understand about the current distribution of *Alviniconcha* species in the SW Pacific, these may also belong to one or more of the *Alviniconcha* species from the current study (either two phenotypes of a single species or more likely, a morphotypes each from two different species). However, synonymous *Alviniconcha* juvenile morphotypes are also described from the Alice springs vent site [16], where only *A. hessleri* is known to occur. This suggests that either juvenile morphology is subject to considerable phenotypic plasticity in *A. hessleri*, or larval supply includes more than one species but post-settlement processes favour *A. hessleri*. More juvenile samples are clearly needed to elucidate whether it is site-specific larval supply or post-settlement processes are driving adult distributions.

Periostracal hair arrangement might prove a useful tool for identifying other *Alviniconcha* species. The relatively high-resolution shell photos of each of the six described species of *Alviniconcha* that appear alongside their molecular diagnoses [1] provide an opportunity to make speculative comparisons informed by data from the current study. In that paper the authors allude to plasticity in both the density and arrangement of the periostracal bristles and spire height in the genus, exemplified by their shell photos (i.e. Fig. 2 in the Johnson et al. 2015 study [1]). However, since the most extreme examples of these trait differences were in museum specimens that were not sequenced with success, they were not able to exclude the possibility that these differences might be related to different species, rather than inter-individual variability within species (e.g. some *Provanna* species [37]). Unfortunately, for several of the new species described in [1], only partial soft tissue samples were available (e.g. *A. kojimai*, *A. strummeri, A. boucheti*). In other species, morphological conclusions could not be made (e.g. *A. adamantis and A. marisindica* in [1]). However, close examination of these photos reveals that the shell of *A. adamantis* pictured, like *A. strummeri*, possesses bristles of uniform length and yet, like *A. kojimai*, has clearly identifiable inter-bristle periostracal ridges, albeit more rounded. *Alviniconcha marisindica*, like *A. boucheti*, appears to possess a somewhat similar bristle arrangement in the shell pictured, with an extra very short bristle between longer bristles in a 1°- 3°- 3°- 2°- 3°- 3°- 1° pattern. Finally, *A. hessleri* may possess a bristle arrangement that superficially resembles that of *A. kojimai*, though bristles are densely packed and difficult to discern with certainty in the *A. hessleri* shell photo [1]. Evidently further samples in which periostracal bristle arrangements and columellar morphology can be matched against molecular assignments are needed, before broader statements concerning diagnostic traits can be made.

Few data are available regarding the soft-body anatomy of *Alviniconcha*. Findings in the current study agree for the most part with those presented by Warén and Bouchet [3], which we now believe to have been largely based on *A. kojimai* and *A. boucheti* specimens. Placing specific shell characteristics aside, we find that *Alviniconcha* morphology and anatomy described in [3] pertaining to the organisation of the animal and in particular to the alimentary, circulatory and excretion systems remain valid at genus level. This is based on the evidence from our study that soft-body characters are highly conservative in SW Pacific species and possibly the genus, based on the synonymous external anatomy of *A. marisindica* presented in [9], the sixth *Alviniconcha* species found at vent sites along the Central Indian Ridge. The notable exception in that study is the labelling of the gonad, which suggests it is located at the posterior-most region of the animal. However, anatomically the *A. marisindica* specimen strongly resembles *A. boucheti,* its most closely related congener. A bulge in the anterior pallial floor clearly visible in that photo is very suggestive of a gonad location akin to that described in the current study and [3], in the posterior cephalopedal haemocoel. The digestive gland (labelled “dg”, in that study) is identical in appearance to the anterior digestive gland in the current study, suggesting that the organ labelled “g” (i.e. gonad) is in fact the posterior digestive gland.

Investigation into the nervous system in the current study was restricted to the identification of the circumoesophageal nerve ring, some visible nerves and the putative location of the main ganglia found in the buccal cavity. We did not attempt to confirm whether these are true ganglia histologically, however their location suggests an epiathroid nervous system. The circulatory and excretory systems, including the locations of the auricle and ventricle in relation to the nephridium, agree with accounts in [3] and are very similar to other provannid species, such as *Provanna lomana* Dall, 1918 [38]. The muscularised nature of the ventricle suggests blood is actively pumped, from the ctenidium to arterial sinuses throughout the body. However, the relative volume of the heart (0.5% of soft-body volume) is ten-fold smaller than that of *Chrysomallon squamiferum* C. Chen, Linse, Copley & Rogers, 2015, a peltospirid vent gastropod with a giant heart (4% of body volume, [39]) and symbiont-housing oesophageal gland [27]*.* Many parameters can affect heart morphology and function: metabolic demands, peripheral resistance, haemocyanin concentrations and properties in the haemolymph. Metabolic demands for this symbiosis have been shown to be quite high compared to other gastropods [18, 40] but additional studies are needed tease out these various contributory factors.

Certain aspects of the reproductive system in [3] however, do not entirely agree with findings in the current study. Warén and Bouchet describe several blind-ending sacks or loops visible externally, containing iridescent sperm, located in the proximal region of the gonoduct (within the gonads of presumptive females), which they identify as seminal receptacles. This is mentioned in the broader context of having found a small number of egg capsules at two of the sampling sites that were thought to be from *Alviniconcha* (based on the fact they were not from *Ifremeria nautilei* Bouchet & Warén, 1991, another abundant co-occurring provannid). However, tissue squashes from gonads that they performed were mostly inconclusive. In the two (female) specimens that were sexed by this method, they do not state whether the gonads of these specimens possess this feature. A receptacle of this sort – the description of which bears a striking resemblance to the branching structure found in *male* gonads in the current study – was not found in any females examined. Although these females were exclusively frozen specimens, such an obvious reproductive feature would be difficult to overlook. In addition, gonads in *Alviniconcha* are unusual in that white, creamy gonads are female and red/orange gonads are male (the opposite is often true in molluscs). It is therefore possible that the structures described in [3] are in fact the terminal branching ducts that we have observed in males (also replete with mature spermatozoa) and that the aforementioned context and atypical gonad colouration has led to the conclusion that females possess seminal receptacles. New, appropriately fixed female specimens (ideally with ripe gonads and a complete gonoduct) are needed to clarify this, however.

The hypertrophied ctenidium of *A. kojimai* and *A. strummeri* occupies around 10–11% of the total soft-body volume and as such, is unusually large for the Provannidae (except *I. nautilei* [41]), reflecting their association with branchial, bacterial symbionts [9–15]. Although no measurement of volume was possible for *A. boucheti,* the ctenidium of this species was similar in size, relative to the animal. These percentages are in good agreement with percentage gill-tissue mass/total-tissue mass of 10.2% calculated previously for Lau-Basin *Alviniconcha* species (species was/were not identified), but lower than that calculated for *I. nautilei* [18]. As in [3], we found that *Alviniconcha* lacks a pallial tentacle or a hypobranchial gland (found in several smaller-sized provannid species [38, 42, 43]). The arrangement of branchial vessels (both primary, secondary, efferent and afferent) and the organisation of filaments described in the current study corroborates previous studies examining several species from this genus (*A. kojimai*/*boucheti*, most likely, in [3] and *A. marisindica* in [9]). Unlike *Provanna* species (e.g. [38]), *Alviniconcha* does not appear to have an oesophageal gland associated with the mid-oesophagus. The stomach is greatly reduced relative to the size of the animal (< 0.05% of soft-body volume = 15–20 mm^3^ at SH 69.9 and 72.3 mm, N.B [3]. estimated stomach of 10 mm^3^ at SH 45 mm), particularly in comparison to provannids not engaged in chemosymbiosis [38, 43], whose stomach volumes are around 100 times larger relative to total body volume [3]. Thus although radulae of all three species provide evidence that grazing takes place, the relative volumes of the symbiont-bearing ctenidium versus the stomach and the dominance of mucus in a simplified alimentary system both indicate that most of their nutrition is likely to depend upon symbioses.

The mechanism by which symbionts contribute to nutrition in *Alviniconcha* remains poorly understood. However, all three species produce substantial quantities of mucus (predominantly branchial), a fact discovered during dissections independent of fixation approach (though frozen specimens stood out in this regard). Filament cleaning demands in a ctenidium of this size likely necessitate such quantities. As in previous studies (e.g. [3]), mucous strings in both frozen and preserved specimens were frequently present in the neck groove leading from the right (exhalant) region of the pallial cavity to the extended anterior flap of the epipodial fold, directly right of the eminently extendable snout. Given the energetic costs of producing mucus are high [44], it is possible that the neck furrow acts as a conduit to deliver mucus to the mouth for recycling. Ctenidium-derived particles immobilised in this mucus may deliver supplementary nutrition [44]. Some of this branchial mucus might even contain bacterial symbionts (e.g. like the bacteria-rich branchial mucus sloughed by *A. marisindica* during periods of low-temperature-induced stress [40]), representing one means by which symbionts could be exploited as food.

Another avenue may be through the lysosomal digestion and assimilation of bacteria within filaments (as suggested in [15]). Preliminary histological evidence presented in the current study suggests that lysed, organic material is being transported directly from gill filaments towards secondary efferent branchial vessels, and thus to the primary efferent branchial vessel. Further study is needed to ascertain from where exactly this debris originates (e.g. transport of autotrophically fixed carbon by-products released by gill-associated symbionts, or remnants of symbionts digested by hydrolytic endocytosis [45]).

The current study was also undertaken to assess to what degree anatomical adaptations might contribute to habitat partitioning in those *Alviniconcha* species that occur over small spatial scales in the SW Pacific. In the Manus, Lau and North Fiji back-arc basins, *A. kojimai* typically occurs in comparative proximity to *A. boucheti* and much less frequently, *A. strummeri* [prior to this study, vent fields in the Southern Lau Basin only 11]. Only one record exists of *A. strummeri* and *A. boucheti* co-occurring within a single vent field, on separate edifices [“ABE-3 and ABE-4”, 11]. The sample origins of the current study indicate that these species-distribution patterns persist further north and over finer spatial scales and expand the known geographic ranges of all three *Alviniconcha* species to include the Wallis and Futuna volcanic area. Previous studies have suggested that geochemical habitat partitioning of this type is made possible by the metabolic diversity that distinct symbioses provide, inferred from a correlation between inter-site variability in end-member fluid composition and the symbiont composition of host species at each site (e.g. [11]). For example, a study in the Lau basin, found that *A. boucheti* with its campylobacterotal symbionts occurs in dominant numbers at venting sites characterised by higher H_2_ and H_2_S concentrations, while *A. kojimai* and *A. strummeri* with their gammaproteobacterial symbionts occur in greater abundances at fields with lower H_2_ and H_2_S ([11], as seen in free-living forms of the same bacterial classes, [46–48]) where, in cases of co-occurrence on a single edifice, one species is typically very dominant. The authors argue that these host distribution patterns likely reflect distinct metabolic capabilities or physiological needs of their symbionts. A recently confirmed capacity for H_2_ oxidation in *Alviniconcha* campylobacterotal symbionts [25], first suggested by [23], appears to support this hypothesis. This would enable the use of discrete abundant energy resources in a highly limiting spatial environment. This hypothesis assumes that the hosts lack species-specific adaptations that might facilitate congeneric niche partitioning such as differences in anatomy that might indicate differing tendencies or capacities to ingest and assimilate food (e.g. markedly different radular or stomach morphology), or the presence of novel adaptive tissues or organs not evident from gross morphology. Having examined the morphology of these species in detail, it is evident that *A. kojimai*, *A. strummeri* and *A. boucheti* display highly conservative functional anatomies across all three species, providing support for the symbiont-function (and/or host physiology) hypothesis from an adaptive evolutionary viewpoint. A recent genomic study of symbiont bacterial partners of *I. nautilei*, *A. kojimai*, *A. strummeri* and *A. boucheti* has identified little evidence in their genomic content for distinct symbiont functional profiles [24], suggesting differences relating to gene expression and regulation (e.g. [23]) and/or differences in host physiology may mediate habitat partitioning. The demonstrated use of partially oxidized forms of sulphur for chemoautotrophy (as an alternative to more reduced sulphur sources found in end-member fluids) by symbionts of several prominent chemosymbiotic vent taxa from the SW Pacific including ‘*Alviniconcha* spp.’, provides tantalising evidence of one mechanism through which interspecific competition among hosts could be mitigated [13]. Clearly, further analyses of host physiology and symbiont gene expression is needed to better understand the possible contribution of each to maintaining regional patterns in habitat use.

Sampling opportunism and ad-hoc adjustments to sample design are intrinsic to deep-sea biology, as limited time at sea and unforeseeable technical problems typically place constraints on dive time on the sea floor. The deep sea is also still relatively unexplored, so it is not always evident what can be expected from new sampling sites. When specimens used in the current study were first collected and preserved, it was not believed possible to differentiate between species. Thus, by sheer happenstance, all specimens of *A. boucheti* collected were frozen, limiting analyses to morphoanatomical examination and restricting histology to more-suitably fixed branchial filaments only. Similarly, all whole specimens fixed in formalin intended for analyses that required greater tissue preservation happened to be *A. kojimai*, with ethanol-fixed samples including both *A. kojimai* and *A. strummeri*. Since the current research now provides a means to discriminate SW-Pacific *Alviniconcha* spp. by sight and with a high degree of reliability, similar problems should be overcome in this region for these species, allowing more strategic sampling and experimental designs with testable hypotheses, defined a priori.

The current study took advantage of a standard CT scanner to elucidate detailed anatomy almost impossible to establish by more traditional approaches, on samples already fixed for other techniques. At no point was sample treatment intended for CT analyses. Consequently, specimens fixed by wildly different approaches were used for CT scans. Of these, perhaps unsurprisingly, CT-scans from the 4-% formalin fixed specimen of *A. kojimai* were the most fruitful in terms of tissue detail and the easiest to segment semi-automatically, due to marked greyscale contrast between tissues (Additional file 4a). Some tissues in the alcohol fixed specimen of *A. strummeri* were not well resolved, particularly those typified by high-lipid content, such as the gonad (at least in females) and digestive glands. In addition, it is likely that tissue shrinkage was quite severe in this specimen, as transfer by incremental alcohol concentrations was not used during preservation [49]. However, comparisons of shrinkage severity across fixation approaches are confounded by specimen size and species. The frozen post-fixed *A. boucheti* sample, while not at all suited to segmentation, was still of sufficiently high quality to identify organs and thus corroborate observations made during dissections. It is for reasons of variable shrinkage that CT-derived organ volumes in the current study are generally expressed as proportional values relative to soft-body volume. This allows comparisons across species and methodologies (e.g. dry-mass ratios [18]). In instances where the degree of shrinkage in a given tissue type is greater or less than the average shrinkage across the soft body as a whole, relative volumes may be slight under- or overestimates (respectively). Such analyses could have been improved further by the implementation of 1) suitable pre-treatment and contrast-staining protocols (examples in [49]) to minimise shrinkage and potentially increase heterogeneity in tissue contrast making tissue segmentation far easier to automate and; 2) more powerful imaging approaches (e.g. synchrotron imaging, [50]) which would yield faster, higher resolution data, reducing the need for complimentary histological analyses. Despite this, a wealth of information could already be gleaned from performing the segmentation of volume data generated from the CT scans, even for *A. strummeri*, fixed directly in 96% ethanol. As new techniques develop to image organisms at finer spatial scales, these techniques will no doubt become more widespread. Such approaches are already being used extensively to rapidly digitise museum specimens worldwide. These initiatives are not only generating new data (e.g. [51]), they also provide an invaluable means to ‘back-up’ samples of paramount importance to our natural heritage, such as those tragically destroyed in Brazil’s National Museum in Rio de Janeiro in September 2018 [52].

## Conclusions

The current study has identified certain distinguishing characteristics that may be used to discriminate between *A. kojimai*, *A. strummeri* and *A. boucheti* (resulting in amended diagnoses for all three species) and strengthens the hypothesis that the regional co-occurrence of certain *Alviniconcha* species is likely driven by something other than anatomical adaptations, perhaps through differences in host physiology or in the functional niches of symbiont assemblages. The authors hope that the results from this study may be used to inform future studies on these and related species of chemosymbiotic gastropods and demonstrates the utility of 3D imaging techniques in answering anatomy-informed questions.

## Methodology

### Aims of study

The current study seeks to determine whether morphological characters yet to be documented could be used to discriminate the three congeneric gastropods *A. kojimai*, *A. boucheti* and *A. strummeri* and whether these (or other) interspecific differences identified during comparative functional anatomical analyses might provide evidence for drivers of habitat partitioning at the organismal level.

### Sampling and specimen treatment

Samples were collected using the HOV Nautile from four venting sites within the newly discovered Fatu Kapa vent field (Stéphanie, Aster’X, Obel’X and Fati Ufu, Fig. 1) during the FUTUNA-3 cruise in May–June 2012 (DOI:10.17600/12010040). Over a hundred specimens of *Alviniconcha* were collected, distinguished from other gastropod taxa based on gross morphology. Most were frozen at − 80 °C for molecular analyses but three were fixed in 96% ethanol and six in 4% formalin (subsequently transferred to 96% ethanol). Following species identification using molecular techniques and some general observations regarding shell morphology, a subset of these samples was used for detailed anatomical study in the current study.

### Species identification and shell characteristics

Genus was assigned based on morphological observations, after which total DNA was extracted for COI barcoding (Additional file 7). Resulting sequence chromatograms were visualized, assembled and edited using Geneious v.10.0.5 [53]. Finally, sequences were matched to publicly available databases using the NCBI blastn algorithm [54]. All sequences are deposited in GenBank (Table 1).

### Dissections

Dissections were performed using heat and UV-sterilised tools on frozen (*n* = 28, all species), formalin- (*A. kojimai* only *n* = 2) and ethanol-fixed specimens (*A. kojimai n* = 1, *A. strummeri n* = 2) by first removing the shell and then dissecting and separating organs and tissue samples for further investigation. Ethanol- and formalin-preserved adults were initially dissected based on the limited anatomical data available from similar species [2, 3] with subsequent, more meticulous dissections following preliminary histological analyses. Certain regions were targeted; 1) the ctenidium (gill) and enveloping pallial epithelium (which includes the rectum); 2) the buccal mass (i.e. dorsal folds, salivary ducts, odontophore and contractile musculature, radula and radular sac); 3) oesophagus, salivary glands and granular cephalopedal tissue; 4) the gonad (when present); 5) lower posterior oesophagus, the stomach, and associated digestive glands; 6) the cardio-renal complex (i.e. heart, nephridium); 7) the remainder of the body (mainly muscle, nervous and vascular tissue). Photographs and detailed sketches were compiled during several dissections. Tissue smears were performed on frozen gonads and when present, oocyte diameters were measured under a compound microscope.

### Scanning electron microscopy (SEM)

Radulae were gently teased out from the anterior of the buccal mass from three frozen specimens of each species. Tissue was removed by digestion using proteinase K and the tissue-free ribbons were dried and mounted on adhesive tape according to Moretzsohn [55]. Mounted radulae were placed on stubs, critical-point dried (LEICA EM CPD300) and coated by gold-sputtering (~ 10 nm thick, Balzers Union SCD040). SEM was performed on a FEI QUANTA 200 electron microscope.

### Histology

Preliminary dissections were used to determine which organs would be investigated histologically and from where the tissue samples would originate in each. Resin choice depended on tissue sample size (large being > 2-mm cutting diameter, embedded in PEG stearate; small tissue was embedded in LR white, see Additional file 7). PEG-embedded tissue was stained using standard histological protocols for Harris’ Haematoxylin (aqueous) and Eosin-Y (in alcohol). Semi-thin sections of LR-white embedded tissue required extended staining times and the use of aqueous Eosin-Y (after [56], see Additional file 7). Slides were viewed and photographed under a Zeiss AxioImager-Z2 microscope. Multiple micrographs were captured (Zeiss AxioImager.Z2) and stitched to form image mosaics using the Tiles module in the associated microscope software Zen 2 (Blue edition). Histological results later informed more detailed dissections.

### CT-scanning: reconstruction, data analyses and visualisation

A specimen of each species was selected for CT scanning (formalin-fixed *A. kojimai*, preserved in ethanol; ethanol-fixed *A. strummeri* and; a frozen *A. boucheti*, post-fixed in 96-% ethanol while being defrosted at 4 °C). Each was infiltrated with 1%-Lugol’s iodine (I_2_/KI) contrast stain in ethanol over 14 days, beginning with serial transfer from storage conditions (increasing stain concentration by 0.25% day^− 1^ over first 3 days). Samples were rinsed 3 times for one minute in absolute ethanol prior to being placed in a Nikon HMX CT-scanner and immobilised with sponge in a sealed bag. Scans were carried out using 3000+ projections (number of images taken during rotating acquisition through 360°), at the highest magnification that allowed the whole soft body to remain in the field of view (parts of shell were sometimes excluded from scan). The projection data was aligned and registered as a voxel matrix: effectively a rasterised 3D map of signal, where pixel values ultimately define voxel greyscale data. This data was transformed into a RAW image stack.

Raw image stacks were converted into a series of 8-bit greyscale images (henceforth ‘volume’) in Fiji (Image-J), based on the associated metadata generated during the scan. Initial and final image slices containing no tissue were excluded to reduce subsequent memory demand of volume manipulation. Volumes were then imported into the open-source 3D rendering software Slicer (v. 4.10). For details on software application see Additional file 7 and Figs. 1, 2, 3, 4 within. Physical dimensions of volumes were specified by imputing scan-specific imaging resolutions as scaling factors, obtained from scan metadata (i.e. the size in microns of each pixel and the virtual thickness of the image slices, together constituting the dimensions of the voxel data). Resolutions were between 29 and 37 μm^3^ voxel^− 1^. Volumes were first visually interrogated by rendering voxel data in 3D directly. To create anatomical models, 3D regions corresponding to different tissue types were mapped from 2D areas assigned to different tissues in each CT slice in the volume, using a variety of manual and semi-automated tools (Additional file 7). Tissue identification during this ‘segmentation’ process was informed by knowledge gained from dissections and histology. Surface-mesh models were generated and exported from the segmentation as .*stl* files and imported into Paraview v. 5.5 to create anatomy figures by colour-coding segments, increasing transparency where necessary and adjusting lighting to optimise 3D rendering. Interactive *U3D*-type models suitable for embedding in a PDF using Adobe Acrobat, were compiled by first reducing the complexity of surface-mesh models in Paraview (by constraining the number of polygons per mesh with the ‘decimator’ filter), then saving each surface mesh as an individual .*obj* file. These were imported into DAZ studio (v. 4.10), where identical colour-coding and transparency settings to those used in 2D figures were implemented. After adding suitable lighting, the model data were exported as a single. U*3D* file. All figures were compiled in Inkscape v. 0.92.

Of the three specimens scanned using computed tomography, only *A. kojimai* and *A. strummeri* were processed by segmentation (to create anatomical models). This is because in addition to some tissue distortion, CT-derived volume data for *A. boucheti* suffered from very narrow threshold ranges across tissue types, making automated segmentation infeasible. However, just as in the dissections performed on this species, careful examination of individual reconstructed slices throughout the *A. boucheti* volume revealed its anatomy to comparable to that of *A. kojimai* and *A. strummeri*.

## Supplementary information

**Additional file 1. **3D visualisation – with interactive model – of gross anatomy in *Alviniconcha kojimai*. Larger version of the CT-model schematic in Fig. 4a depicting the gross anatomy of *A. kojimai*. Embedded within is a more detailed 3D anatomical model that includes additional data not presented in the 2D schematic. *N.B. ganglia depicted in the interactive model are putative and not confirmed histologically.*

**Additional file 2. **3D visualisation – with interactive model – of gross anatomy in *Alviniconcha strummeri.* CT-model schematic of *A. strummeri* depicting the gross anatomy. Embedded within is a more detailed 3D anatomical model that includes additional data not presented in the 2D schematic.

**Additional file 3. **Specimens of *A. kojimai* with and without last shell whorl. Photographic plate of gross anatomy, like that of Fig. 2, but for *Alviniconcha kojimai*.

**Additional file 4.** Appearance and quality of CT volumes for each species, given separate fixation regimes. CT volume screenshots summarising the appearance and utility of the three specimens scanned, considering differing preservations regimes used for each species. Also provides further evidence concerning the internal morphology of columella.

**Additional file 5. **Additional morphological features identified during dissections (*A. kojimai*). Photographs that identify additional anatomical details that were not core to the manuscript but merit mention. Includes a photo of the neck furrow when twin channels are present; the anterior-most ventral face of the branchial axis where the osphradium can clearly be seen, the adapical face of the visceral hump where the visceral blood sinus is located and; mosaic micrographs of branchial filaments taken from each *Alviniconcha* species.

**Additional file 6.** Examination of jaws and anterior radula. Scanning-electron microscopy images of one of the paired jaws and of the anterior-to-posterior comparison of the radula, providing evidence that it is actively being used.

**Additional file 7.** Supplementary methodology (and methodological figures therein). Additional information on the histological approaches used in the current study and some details concerning the segmentation process in the software 3D Slicer, used to create 3D models from computed tomography data.

## Data Availability

Image volume data analysed in the current study, generated from CT scans, are available from the corresponding author on reasonable request. Voucher specimens used in CT analyses are deposited in the Muséum National d’Histoire Naturelle in Paris, France (see amended species descriptions for specimen vouchers).

## Abbreviations

1°, 2°, 3°: Primary, Secondary, Tertiary (respectively)
BL: Bristle length
CT: Computed tomography
EEZ: European economic zone
HOV: Human-operated vehicle
RL: Radular length
RL_max_: Maximum radular length
SH: Shell height
SW: Shell width
1° ABV: Primary afferent branchial vessel
1° EBV: Primary efferent branchial vessel
2° ABV: Secondary afferent branchial vessel
2° EBV: Secondary efferent branchial vessel
AA: Anterior aorta
ADD: Anterior digestive duct
AOe: Anterior oesophagus
Ap: Apex
ARV: Afferent renal vein
Au: Auricle
BC: Buccal cavity
BM: Buccal mass
BR: Bacteriocyte region
BS: Blood sinus(es)
BV: Blood vessels
CBS: Co-marginal blood sinus
CM: Columellar muscle
Co: Columella
CG: Cerebral ganglia (putative, Additional file 1 only)
CoC: Circumoesophageal complex
CpV: Cephalopedal Vein
Ct: Ctenidium
CT: Cephalic tentacles (except figures 5 and 7, where it is Connective tissue)
DD: Digestive diverticula
DF: Dorsal fold
DG: Digestive gland
EL: Epithelial layer
Ft: Foot
Gd: Gonoduct
Go: Gonad
GS: Gastric shield
GT: Granular connective tissue
H: Heart
HF: Head-foot
In: Intestine
LPN: Left pallial nerve
LTN: Left tentacular nerve
MaT: Major typhosole
MiT: Minor typhosole
Mo: Mouth
MOe: Mid-oesophagus
mPOe: Mid-posterior oesophagus
Mu: Mucosa
Ne: Nephridium
NF: Neck furrow
NR: Oesophageal nerve ring
Od: Odontophore
Oe: Oesophagus
OeP: Oesophageal pouches
Op: Operculum
P: Periostracum (bare)
PA: Posterior aorta
PBN: Pedal blood network
PBr: Periostracal bristles
PBV: Pallial blood vessel
PC: Parietal callus
PDD: Posterior digestive duct
PeG: Pedal ganglia (putative, Additional file 1 only)
PF: Pallial fringe (except in Figure 9, where it is Primary folds)
PG: Pleural ganglia (putative, Additional file 1 only)
PNN: Pedal neural network
POe: Lower posterior oesophagus
PS: Pallial skirt
R: Radula
RSa: Radular sac (Additional file 2 only)
Re: Rectum
RP: Renal pore
RPN: Right pallial nerve
RS: Rectal sinus
RS (ant): Anterior blood vessels extended from rectal sinus
RTN: Right tentacular nerve
RVC: Right visceral connective
SA: Sorting area
SD: Salivary ducts
SG: Salivary glands
SM: Shell margin
Sn: Snout
SN: Siphonal notch
SS: Style sac
St: Stomach
uPOe: Upper posterior oesophagus
V: Ventricle
VM: Visceral mass
VS: Visceral sinus
