# Supplementary material for: Classical and computed tomographic anatomical analyses in a not-so-cryptic Alviniconcha species complex from hydrothermal vents in the SW Pacific
Source: Front Zool. 2020 May 7;17:12. doi: 10.1186/s12983-020-00357-x (PMC7203863; doi:10.1186/s12983-020-00357-x)
Supplement: Supplementary file 1 — Additional file 1. 3D visualisation – with interactive model – of gross anatomy in Alviniconcha kojimai. Larger version of the CT-model schematic in Fig. 4a depicting the gross anatomy of A. kojimai. Embedded within is a more detailed 3D anatomical model that includes additional data not presented in the 2D schematic. N.B. ganglia depicted in the interactive model are putative and not confirmed histologically. [file 12983_2020_357_MOESM1_ESM.pdf]

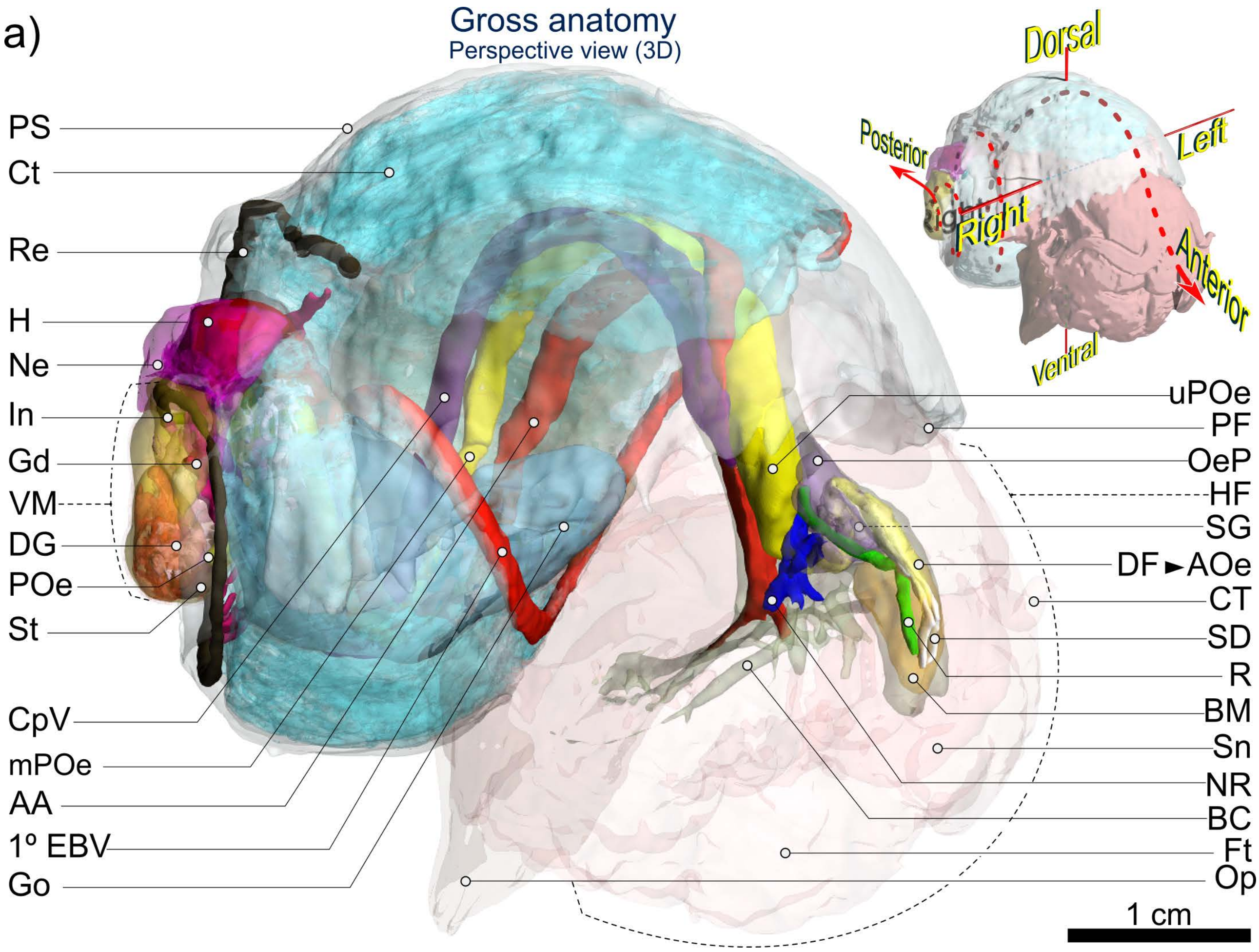

3D visualisation – <u>with interactive model</u> – of gross anatomy in *Alviniconcha kojimai*

This figure is an enlarged version of that in Figure 4a. An interactive model of this specimen is embedded in this figure. To activate, click on the figure and authorise activation of 3D content, then click again to open content. Several example views are provided including a morphological overview as well as a subset of organ/tissue segments that relate to specific functional systems. **The nervous system (partial) is putative and ganglia depicted in the interactive model are not confirmed histologically**. For clarity purposes, some tissues and features present in the interactive model, are not included in the above 3D visualisation. Also, in the model, part of the shell is missing as it did not feature in the scan and two regions of the shell had to be cut for fixation purposes prior to the scan. Abbreviations: **1° EBV** Primary efferent branchial vessel; **AA** Anterior aorta; **ADD** Anterior digestive duct; **AOe** Anterior oesophagus; **BC** Buccal cavity; **BM** Buccal mass; **CpV** Cephalopedal Vein; **Ct** Ctenidium; **CT** Cephalic tentacles; **DD** Digestive diverticula; **DF** Dorsal fold; **DG** Digestive gland; **Ft** Foot; **Gd** Gonoduct; **Go** Gonad; **H** Heart; **HF** Head-foot; **In** Intestine; **mPOe** Mid-posterior oesophagus; **Ne** Nephridium; **NR** Oesophageal nerve ring; **OeP** Oesophageal pouches; **Op** Operculum; **PDD** Posterior digestive duct; **PF** Pallial fringe; **POe** Lower posterior oesophagus; **PS** Pallial skirt; **R** Radula; **Re** Rectum; **SD** Salivary ducts; **SG** Salivary glands; **Sn** Snout; **St** Stomach; **uPOe** Upper posterior oesophagus; **VM** Visceral mass.
