# Supplementary material for: Classical and computed tomographic anatomical analyses in a not-so-cryptic Alviniconcha species complex from hydrothermal vents in the SW Pacific
Source: Front Zool. 2020 May 7;17:12. doi: 10.1186/s12983-020-00357-x (PMC7203863; doi:10.1186/s12983-020-00357-x)
Supplement: Supplementary file 2 — Additional file 2. 3D visualisation – with interactive model – of gross anatomy in Alviniconcha strummeri. CT-model schematic of A. strummeri depicting the gross anatomy. Embedded within is a more detailed 3D anatomical model that includes additional data not presented in the 2D schematic. [file 12983_2020_357_MOESM2_ESM.pdf]

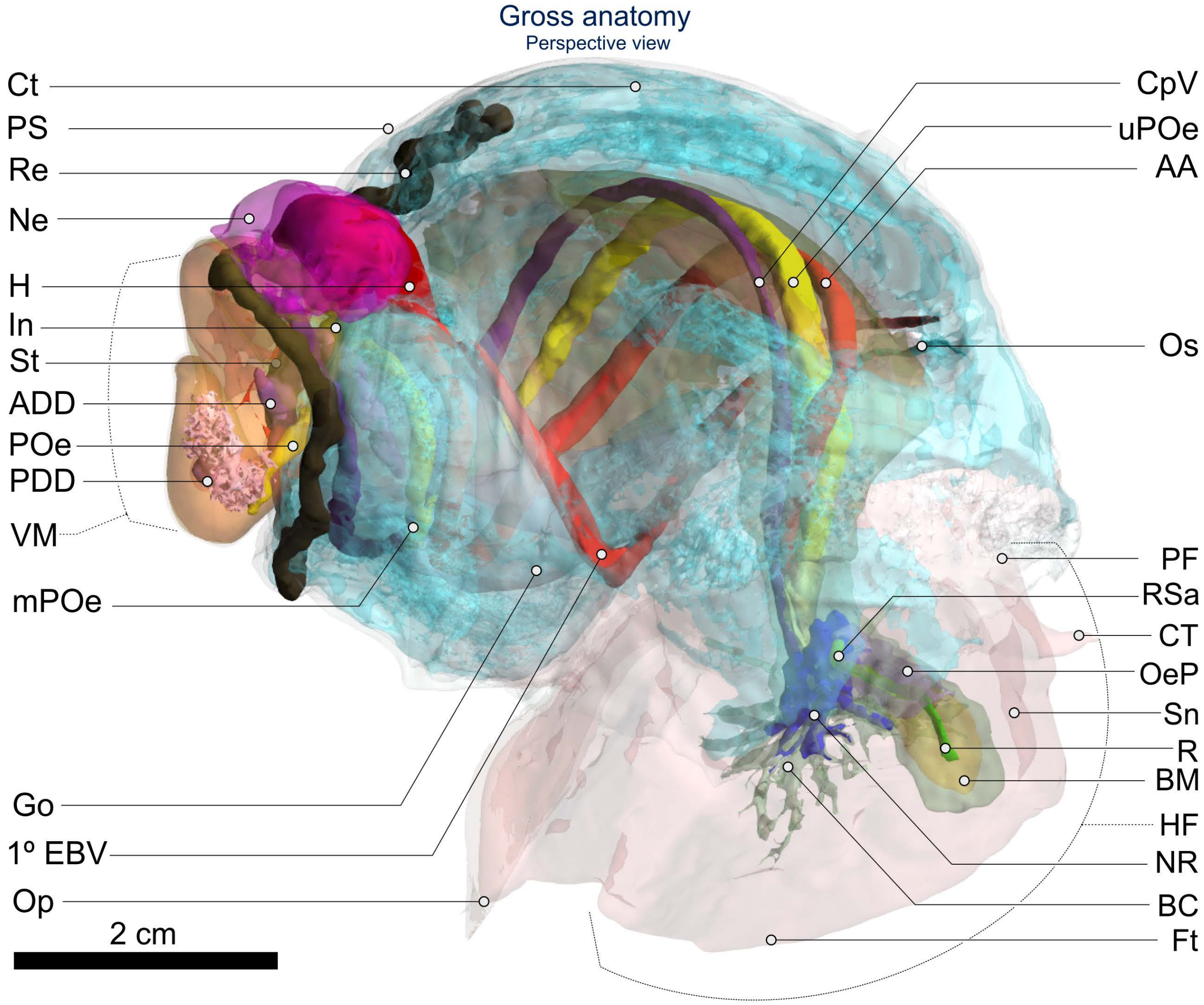

3D visualisation – with interactive model – of gross anatomy in *Alviniconcha strummeri*
An interactive model of this specimen is embedded in this figure. To activate, click on the figure and authorise activation of 3D content, then click again to open content. Several example views are provided including a morphological overview as well as a subset of organ/tissue segments that relate to specific functional systems. Note that for clarity purposes, some tissues and features present in the interactive model, are not included in the above 3D visualisation. Abbreviations: **1° EBV** Primary efferent branchial vessel; **AA** Anterior aorta; **ADD** Anterior digestive duct; **BC** Buccal cavity; **BM** Buccal mass; **CpV** Cephalopedal Vein; **Ct** Ctenidium; **CT** Cephalic tentacle; **Ft** Foot; **Go** Gonad; **H** Heart; **HF** Head-foot; **In** Intestine; **mPOe** Mid-posterior oesophagus; **Ne** Nephridium; **NR** Nerve ring; **OeP** Oesophageal pouches; **Op** Operculum; **Os** Ospradium; **PDD** Posterior digestive duct; **PF** Pallial fringe; **POe** Lower posterior oesophagus; **PS** Pallial skirt; **R** Radula; **Re** Rectum; **RSa** Radular sac; **Sn** Snout; **St** Stomach; **uPOe** Upper posterior oesophagus; **VM** Visceral mass
