# Supplementary material for: Classical and computed tomographic anatomical analyses in a not-so-cryptic Alviniconcha species complex from hydrothermal vents in the SW Pacific
Source: Front Zool. 2020 May 7;17:12. doi: 10.1186/s12983-020-00357-x (PMC7203863; doi:10.1186/s12983-020-00357-x)
Supplement: Supplementary file 3 — Additional file 3. Specimens of A. kojimai with and without last shell whorl. Photographic plate of gross anatomy, like that of Fig. 2, but for Alviniconcha kojimai. [file 12983_2020_357_MOESM3_ESM.pdf]

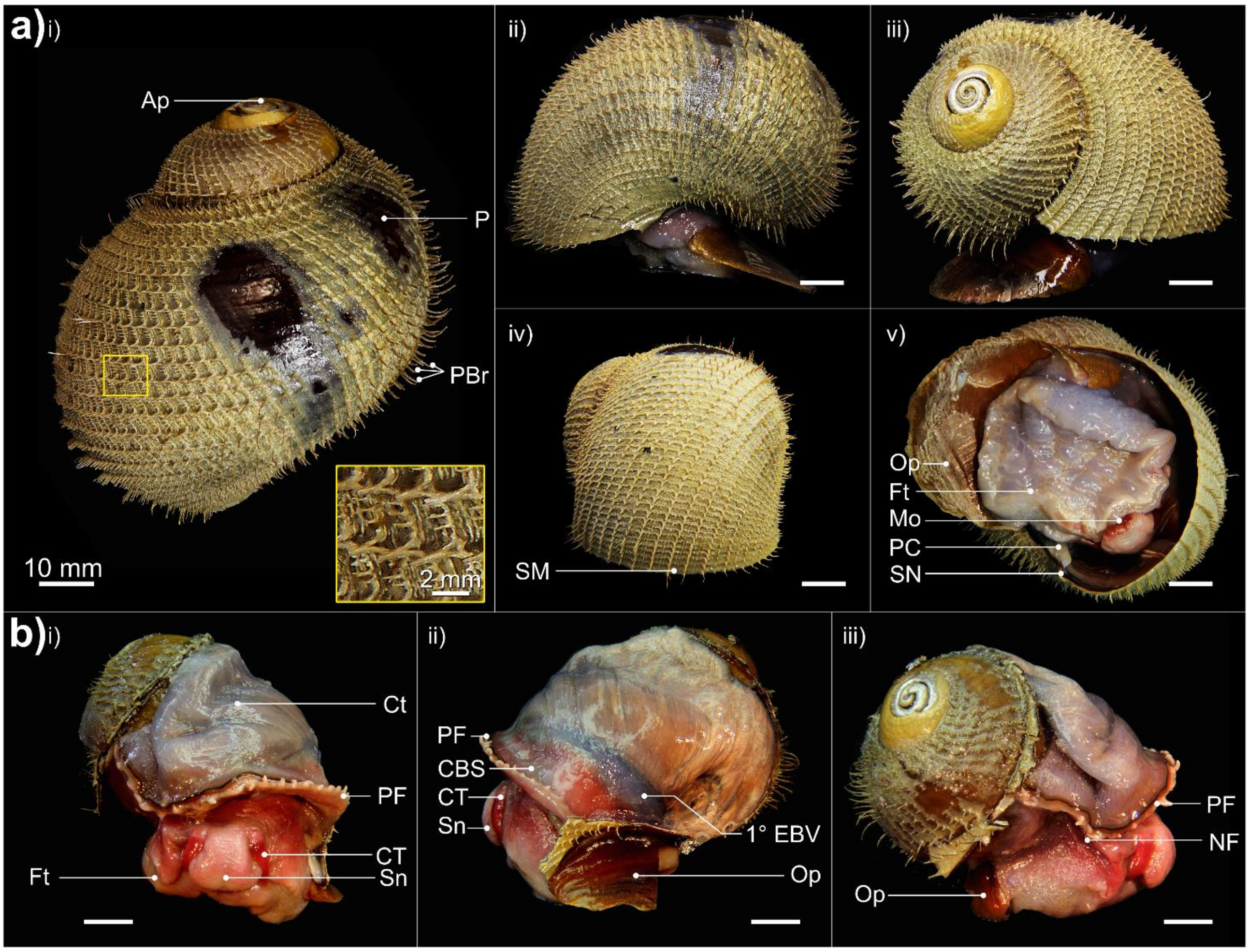

Specimens of *A. kojimai* with and without last shell whorl

a) i) Abapertural view. Inset in a) i) is close-up view of shell region indicated with yellow square: note the ridges that connect the 1° periostracal bristles both spirally and axially. Remaining views in a) are ii) Lateral-left, iii) Lateral-right, iv) Anterior, v) Ventral. Images in b) are of a different individual after the removal of the last shell whorl; b) i) Anterior view, ii) Lateral-left, iii) Lateral-right. Abbreviations: **1° EBV** Primary efferent branchial vessel; **Ap** Apex; **CBS** Comarginal blood sinus; **Ct** Ctenidium; **CT** Cephalic tentacle; **Ft** Foot; **PF** Pallial fringe; **Mo** Mouth; **NF** Neck furrow; **Op** Operculum; **P** Periostracum (bare); **PBr** Periostracal bristles; **PC** Parietal callus; **Sn** Snout; **SN** Siphonal notch;
