# Supplementary material for: Classical and computed tomographic anatomical analyses in a not-so-cryptic Alviniconcha species complex from hydrothermal vents in the SW Pacific
Source: Front Zool. 2020 May 7;17:12. doi: 10.1186/s12983-020-00357-x (PMC7203863; doi:10.1186/s12983-020-00357-x)
Supplement: Supplementary file 4 — Additional file 4. Appearance and quality of CT volumes for each species, given separate fixation regimes. CT volume screenshots summarising the appearance and utility of the three specimens scanned, considering differing preservations regimes used for each species. Also provides further evidence concerning the internal morphology of columella. [file 12983_2020_357_MOESM4_ESM.pdf]

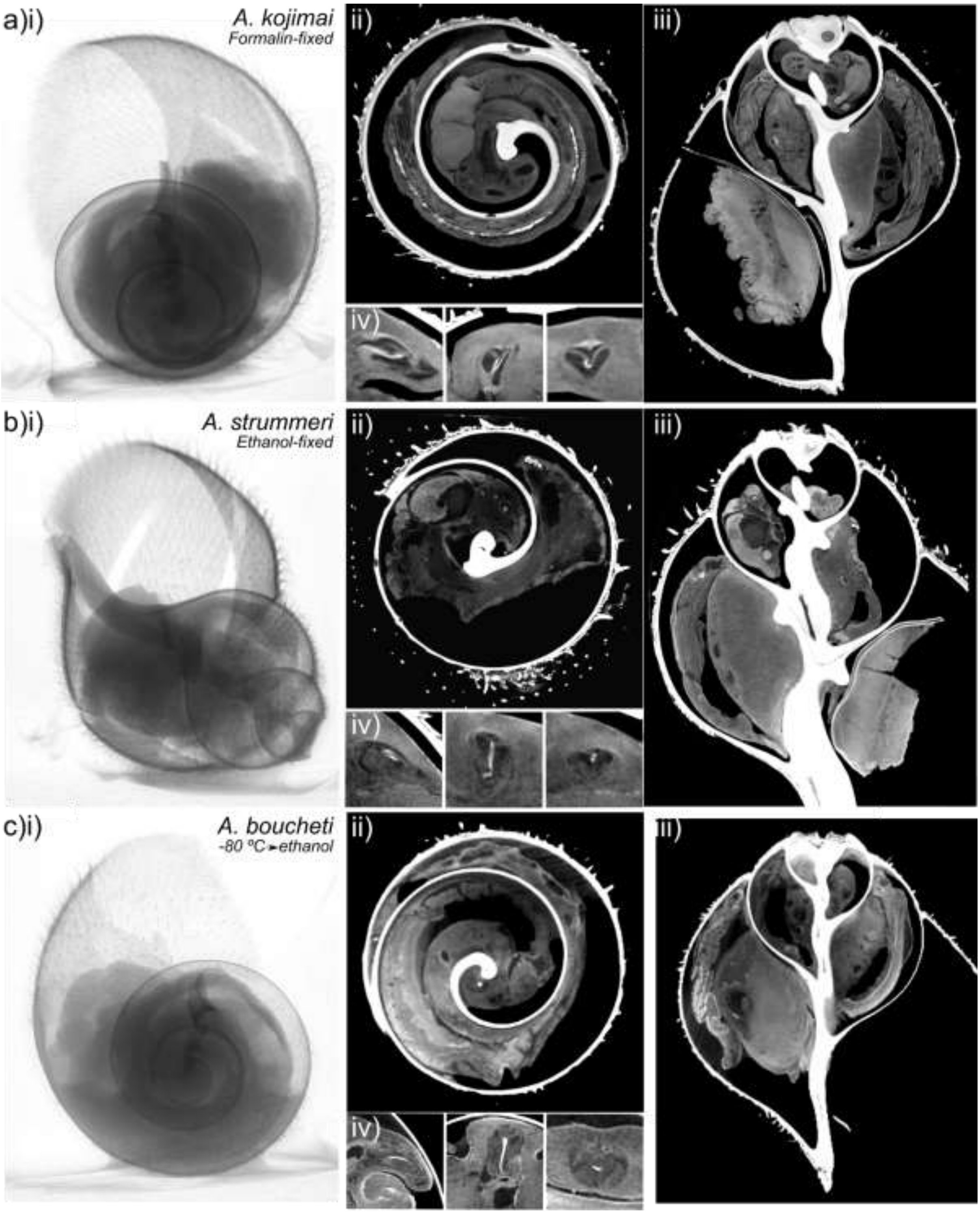

Appearance and quality of CT volumes for each species, given separate fixation regimes
Pictured are a) *A. kojimai* fixed in 4 % formaldehyde and stored in 96% ethanol; b) *A. strummeri* fixed and stored in 96% ethanol and; c) *A. boucheti*, a frozen specimen (-80 °C) that was post-fixed in 96% alcohol under chilled conditions (4 °C) to allow alcohol replacement in thawing tissues. A pre-scan x-ray of each specimen is displayed in (i) and CT volume screenshots of ii) apical slice of the uppermost whorls (spire) including the cardio-renal complex, iii) central axial slice (not in exactly the same dextral position for each species) and iv) sagittal, frontal and transverse slices of the buccal mass. The appearance and utility of the three specimens scanned varied greatly with fixation approach (see main text). Note that the presence of a columellar fold in *A. kojimai* and *A. strummeri* and its absence in *A. boucheti*, can clearly be seen in (ii) and (iii) for each species (repeating bump on columella, where present).
