# Supplementary material for: Classical and computed tomographic anatomical analyses in a not-so-cryptic Alviniconcha species complex from hydrothermal vents in the SW Pacific
Source: Front Zool. 2020 May 7;17:12. doi: 10.1186/s12983-020-00357-x (PMC7203863; doi:10.1186/s12983-020-00357-x)
Supplement: Supplementary file 5 — Additional file 5. Additional morphological features identified during dissections (A. kojimai). Photographs that identify additional anatomical details that were not core to the manuscript but merit mention. Includes a photo of the neck furrow when twin channels are present; the anterior-most ventral face of the branchial axis where the osphradium can clearly be seen, the adapical face of the visceral hump where the visceral blood sinus is located and; mosaic micrographs of branchial filaments taken from each Alviniconcha species. [file 12983_2020_357_MOESM5_ESM.pdf]

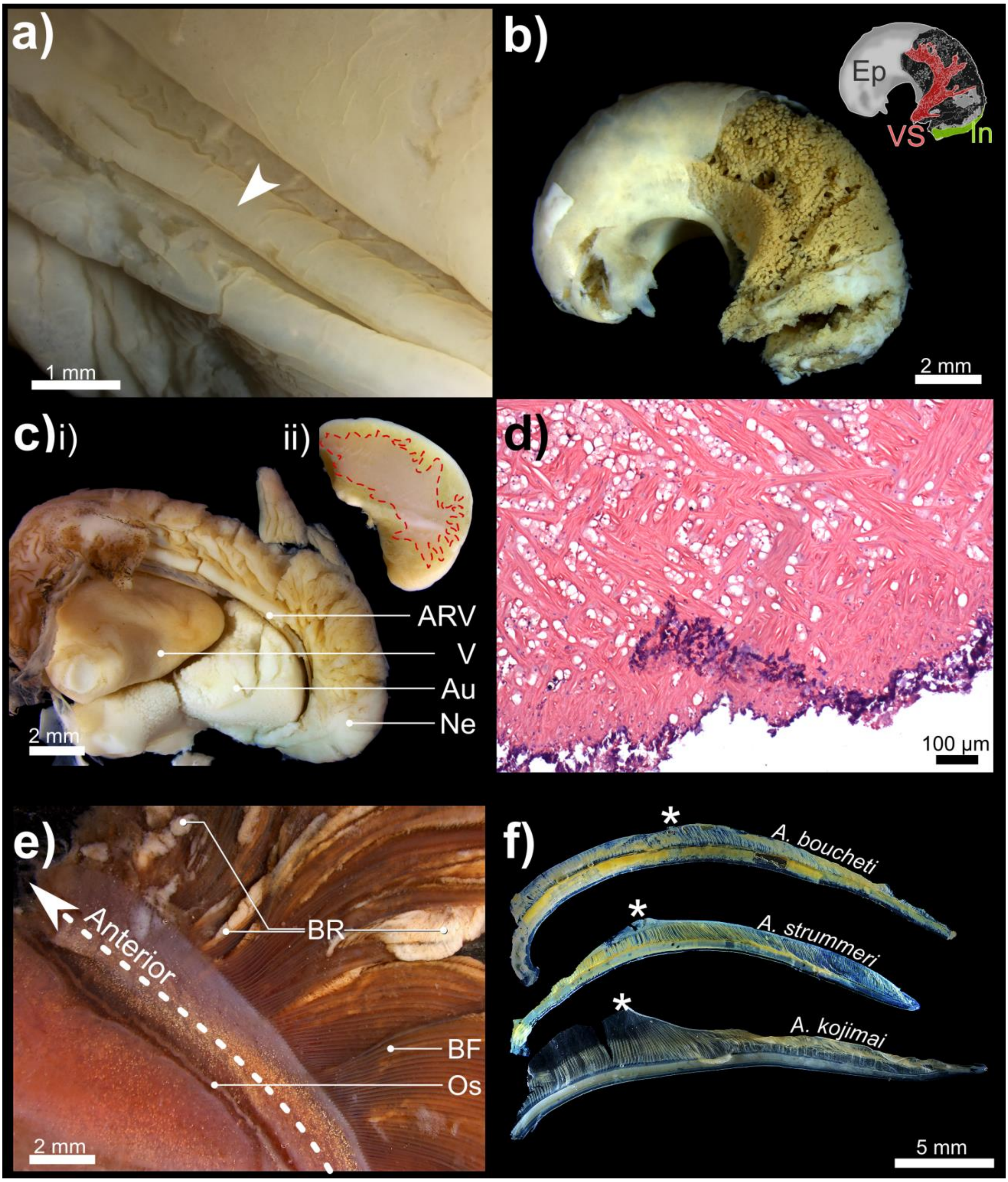

Additional morphological features identified during dissections (*A. kojimai*)

Pictured are a) close-up of a neck furrow with an additional central epidermal fold (arrowhead); b) view of the digestive diverticula visible on the adapical – right – side of the visceral hump, with part of the epithelium removed. The overlying epithelium (**Ep**), branching visceral sinus (**VS,** preserved blood removed to reveal channels) and part of the intestine (**In**) are indicated in the accompanying schematic; c) cardio-renal complex- auricle (**Au**) is characterised by a large pool of preserved blood (similar in consistency to blood found in the primary efferent branchial vessel and anterior aorta, which are both removed). The afferent renal vein (**ARV**) is visible where folds on the right side of the nephridium (**Ne**) have been removed. The inset image (ii) is of the ventricle (**V**) cut in half, with the region where blood and muscle fibres co-occur highlighted; d) histological section of the ventricle wall, showing dense arrangement of criss-crossing muscle fibres (stain: Harris' haematoxylin and Eosin Y); e) location of ospradium (**Os**) in relation to the ventral face of the branchial axis (dotted arrow), from which extend the branchial filaments (**BF**). Note the distinct white spongey appearance of the bacteriocyte-rich region of the filaments (**BR**, displaced ventrally during dissection). These regions were white-to-cream coloured and engorged in this way in *A. kojimai* and *A. strummeri* but were a darker brown and highly mucous in *A. boucheti*; and; f) posterior sides of branchial filaments taken from each of the three species, with *A. kojimai* filaments being particularly broad dorsoventrally (note some proximate filament is missing in *A. strummeri*). The free-floating halves lie right of the asterisks.
