# Supplementary material for: Classical and computed tomographic anatomical analyses in a not-so-cryptic Alviniconcha species complex from hydrothermal vents in the SW Pacific
Source: Front Zool. 2020 May 7;17:12. doi: 10.1186/s12983-020-00357-x (PMC7203863; doi:10.1186/s12983-020-00357-x)
Supplement: Supplementary file 6 — Additional file 6. Examination of jaws and anterior radula. Scanning-electron microscopy images of one of the paired jaws and of the anterior-to-posterior comparison of the radula, providing evidence that it is actively being used. [file 12983_2020_357_MOESM6_ESM.pdf]

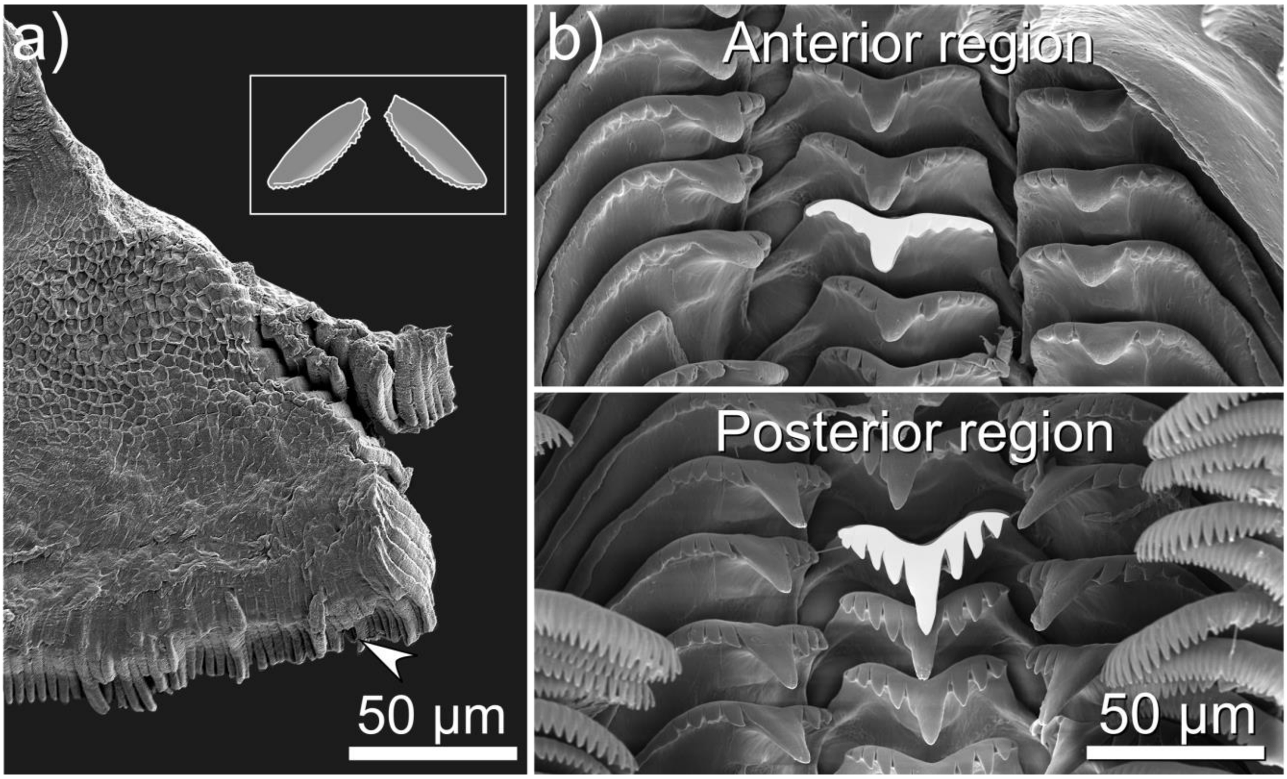

Examination of jaws and anterior radula

Pictured are: a) scanning-electron micrograph of jaw microstructure where the arrowhead indicates cutting edge, with schematic of v-like jaw arrangement in inset (when viewed anteriorly) and b) visual comparison of anterior and posterior radular teeth. Cusps of anterior teeth appear eroded. Note that the anterior teeth also appear yellow-brown in colour when compared with the pristine, transparent posterior region of the radula; both are clearly visible once radula has been teased from odontophore and radular sac (not shown).
