# Supplementary material for: Classical and computed tomographic anatomical analyses in a not-so-cryptic Alviniconcha species complex from hydrothermal vents in the SW Pacific
Source: Front Zool. 2020 May 7;17:12. doi: 10.1186/s12983-020-00357-x (PMC7203863; doi:10.1186/s12983-020-00357-x)
Supplement: Supplementary file 7 — Additional file 7. Supplementary methodology (and methodological figures therein). Additional information on the histological approaches used in the current study and some details concerning the segmentation process in the software 3D Slicer, used to create 3D models from computed tomography data. [file 12983_2020_357_MOESM7_ESM.pdf]

Supplementary methodology

1. Additional details on COI sequencing

DNA was extracted from foot tissue using the CTAB method [1]. Negative controls with reagents only were included for each extraction series. Amplification of a 1228 bp fragment of the Cytochrome Oxidase I (COI) mitochondrial gene was performed using COIf 5'-TCM ACT AAT CAY AAR GAY ATT GGN AC-3'and COIr 5' -CCD CCT AGW CCT ARR AAR TGT TGN GG - 3' primers [2]. PCR mixtures (50 µl final volume) contained 2 ng of DNA template, 0.3 µM of each primer, 0.4 mM of dNTP mix, 2 mM of $MgCl_2$, 1X buffer, and 1.25 U of Promega Taq DNA polymerase. Each PCR assay included a negative control with molecular grade H_2O instead of DNA extract and extraction negative controls to assess for potential contamination. PCR cycling conditions were as follows: 5 min at 94 °C for one cycle, followed by 35 cycles of 1 min at 94 °C, 1 min at 52 °C, and 2 min at 72 °C, and ending with a final extension 7 min at 72 °C. PCR products were sent to GATC Biotech for sequencing of both strands using the PCR primers and two internal additional primers : HCO2198: 5'-TAA ACT TCA GGG TGA CCA AAA AAT CA-3' [3] and COI-3: 5'-GTN TGR GCN CAY CAY ATR TTY CAN GT-3' [4]. Purification and sequencing reactions were performed using the ABI BigDye® Terminator v3.1 Cycle Sequencing Kit (Applied Biosystems, Waltham, MA) and read on an ABI 3730xl sequencer following the protocols supplied by the manufacturer.

2. Histology details

Larger tissue samples were infiltrated in PEG stearate at 45 °C following serial transfer from ethanol to resin (1-hr steps at 1:3, 1:1, 3:1, then four changes of pure resin; the last being overnight). Tissue was then transferred to fresh resin, removed from incubator for tissue orientation and left to set. Blocks were stored at -20 °C. Blocks were trimmed down, ribbons of 7-µm section were cut on a Leica RM2255 microtome and placed on Superfrost plus slides, floating on a large aliquot of Milli-Q water. Following a 10-min relaxation period, excess water was drained.

Smaller tissue samples were infiltrated in LR white (London Resin Co.) at room temperature (3 x 30-min infiltrations, 4th overnight) removing and renewing the resin with micropipettes, following serial transfer from ethanol (1-hr, 1:1). Tissue was then transferred to fresh resin in a gelatine capsule (size 000, Electron Microscopy Sciences, UK), oriented and capped. Polymerisation was at 50°C (20 hours minimum). Gelatine was subsequently dissolved in hand-hot tap water (15 minutes).

LR-white resin pellets were trimmed and wet-sectioned on a Leica EM UltraCut UCT Ultramicrotome (Germany), using a 45° glass knife. Sections (2 µm) were transferred to Superfrost plus slides. Periodic toluidine staining was performed to track progress through tissue (≈10 µm-intervals).

Standard H & E staining was modified for semi-thin sections by extending staining times and excluding ethanol, which distorts LR white. Sections were rehydrated in distilled water (10 min), blotted dry and stained (1 hr) in filtered, freshly acidified Harris' haematoxylin solution (add 5mL glacial acetic acid L^{-1} solution, just prior to use). Slides were 'blued' for 1 min (running-water bath, pH >8). Differentiation, when

required, was performed with 0.5% acetic acid (aqueous). Slides were rinsed (distilled water) to remove high-pH residues, air-dried, stained with 1% aqueous Eosin-Y (pH 5.0, extended stain time of 2.5 hrs), rinsed again (3 x separate 1% acetic acid baths, 1 x distilled water), and air-dried. Sections were cleared (Histoclear II), and mounted immediately (Omnimount©, Electron Microscopy Sciences RT 17997-01, formulated to be used in conjunction with Histoclear II).

3. 3D Slicer platform for CT analyses

3D Slicer (https://www.slicer.org/) is an open-source platform for image processing, analysis and three-dimensional visualization, designed for medical research applications. Input and output formats are many, including DICOM, NIfTI, Meta, NRRD, MGZ but also image sequences, as used in the current study. Volume data are composed of voxels (pixels with a 3rd dimension), and based in physical space (scaling must therefore be provided for CT data formatted as a sequence of images, as the default is 1 mm x 1 mm x 1 mm for one voxel). CT data analysed for the current study was subjected to a processing pipeline prior to segmentation. Pre-processing included: the cropping of background space; re-orientation of volume data using linear transforms; the creation of gaussian blurring filters that when enabled, reduce heterogeneity of greyscale values in physical space (can improve automated segmentation) and; volume rendering, a rapid means to visualise the sample in 3D. Segmentation was performed where possible using automated approaches. Where this was not possible, such as instances where adjacent tissue types have similar greyscale values or when single tissue types display a wide range of grayscale values, manual segment creation was undertaken. Once segmentation was completed, segment statistics were calculated based on volumes and surface areas. Segments were then exported as mesh models for viewing in Paraview (to create 2D figures) and DAZ studio to create the U3D model for embedding in interactive PDF. The following sections provide further details of the segmentation process.

3.1. *Installation of 3D Slicer and getting started*

3D Slicer can be downloaded for Windows, Linux, and Mac **(**https://www.slicer.org/). Nightly builds and stable versions are available. The following applies to 3D Slicer 4.11 (stable version) downloaded for Windows 10. The Slicer community have created a number of useful tutorials and user manuals for each stable release. In addition, a Slicer online forum (https://discourse.slicer.org/) provides a platform for trouble shooting issues with the software.

3.2. *Data import and volume scaling*

Data is imported using >**File** >**Add Data**. To import a sequence of images (NOT an image-stack file, but individual images numbered in the order they ought to appear in the volume), > **Choose file(s) to Add** was used, selecting the first image in a folder containing images to be used in volume only and select > **Open**. Enabling **> Show options** box, and unselecting the > S**ingle file** box loads all images from that folder. Once imported, scaling is defined in the *Volumes* module. If not already selected, the drop-down menu for *Active volume* section can be used to > **Select a volume**. The imported volume should be present (it takes the name

of the first imported image in sequence by default). Once this volume is selected, > **Image spacing** can be defined in the *Volume information* section. This is where the physical scale of each pixel (and thus voxel) is defined based on the ‘resolution’ value from the scan metadata. This scaling is in millimetres.

3.3. *Cropping (resample scalar volume)*

Using the *Resample Scalar/Vector/DWI Volume* extension, the volume can be resampled within a region of interest that encloses the tissue but excludes unnecessary background. More information can be found here: https://www.slicer.org/wiki/Registration:Resampling. It is a useful means to reduce the RAM needed to work with volume data, by reducing the total number of voxels in the volume.

3.4. *Transforms*

Linear transforms are used to re-orientate the three navigating axes of a volume (lower panes in Supplementary methods: figure 1). This permits the specimen to be navigated along axes that facilitate the interpretation of tissue organisation, such as aligned with the spiralling columella and the aperture. Volumes, segmentations and models can be reversibly transformed. Note that some segmentation tools do not work well on temporarily transformed volumes, so may require resampling to create a separate permanently transformed volume. (see 3.3).

3.5. *Preliminary volume rendering*

The volume is visually interrogated by rendering voxel data in 3D, manipulating opacity between a minimum and maximum threshold value for greyscale intensity and so filtering out base background values (Supplementary methods: figure 1). This can sometimes reveal tissue types directly if they have very distinct greyscales ranges (e.g. Blood sinuses / vessels showing as red in Supplementary methods: figure 1). Note that volume rendering runs up considerable CPU activity and occupies several GBs of running memory in large datasets.

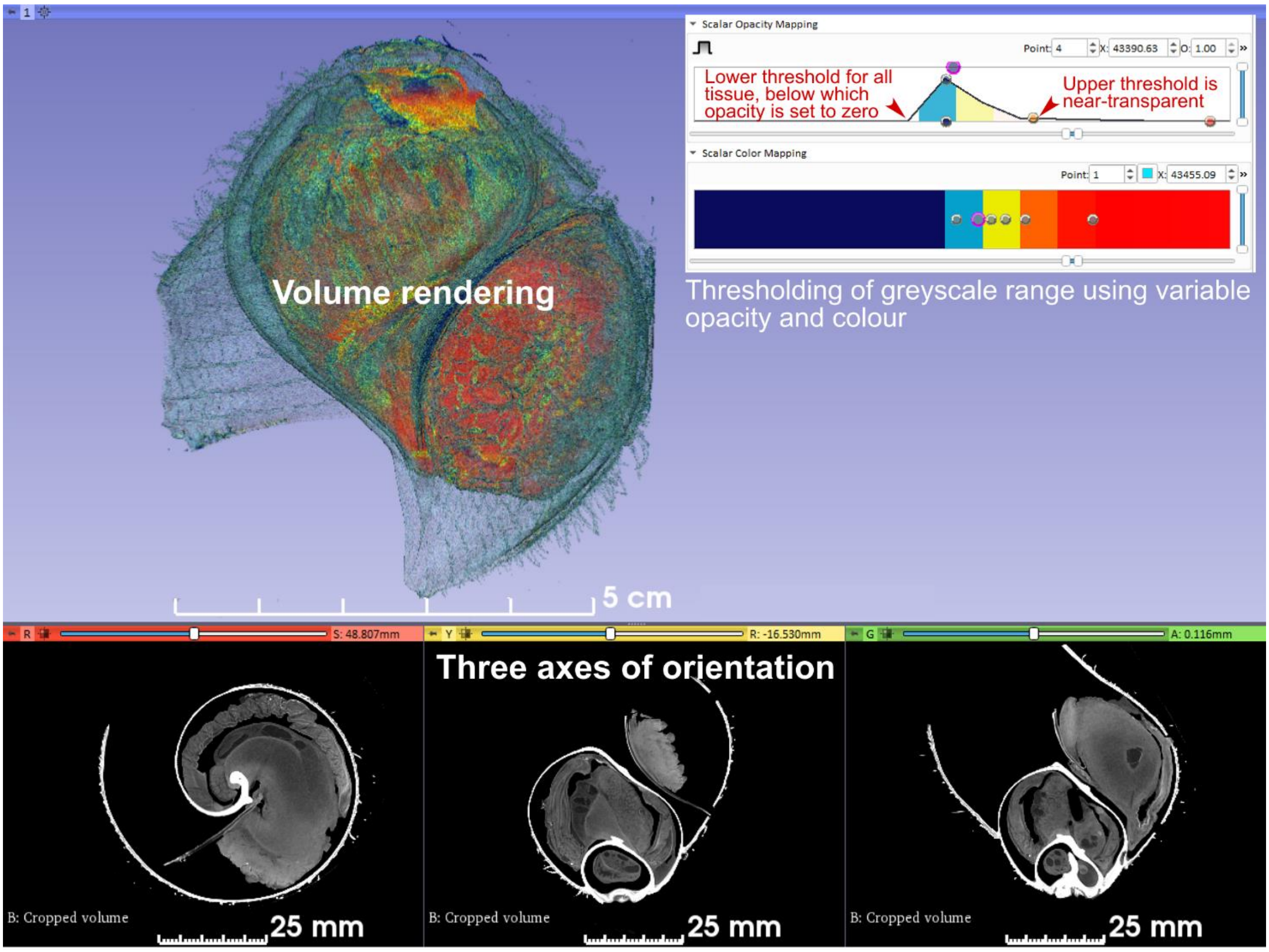

Supplementary methods: figure 1 Volume rendering of *A. kojimai*
Volume rendered in 3D is pictured with the colour-map and opacity settings used for the entire greyscale range of the volume (right inset). Volume data is visualised in three axes of orientation below (conventional view).

3.6. *Segmentation and segment editor*

3D segments (formerly termed labelmaps) are rendered from multiple 2D areas mapped in individual volume slices. Various methods exist to aid in the segmentation of tissues. The following list, though not exhaustive, includes the majority of methods used in the current study. Many have augmented functionality in 3D view.

3.6.1. Paint, draw, erase

Simple tools for manually painting regions of an image. Can be constrained spatially within a greyscale range outside of which pixels are not painted/erased and/or within/outside other segment boundaries (Supplementary methods: figure 2).

3.6.2. Threshold

Selects all voxels that meet lower and upper threshold constraints (Supplementary methods: figure 2, origins of shell segment featured). For organs that have consistent greyscale values, distinct from other tissues, this can be used to rapidly select whole organs (e.g. blood vessels, calcified shell material).

3.6.3. Margin

Used to expand or shrink pre-existing segments (used to refine limits of transparent shell in Supplementary methods: figure 2, following thresholding). Alternating between the two can act to reduce erroneous gaps or extrusions in a segment (contract-dilate technique, see also 3.6.5 *Smoothing*).

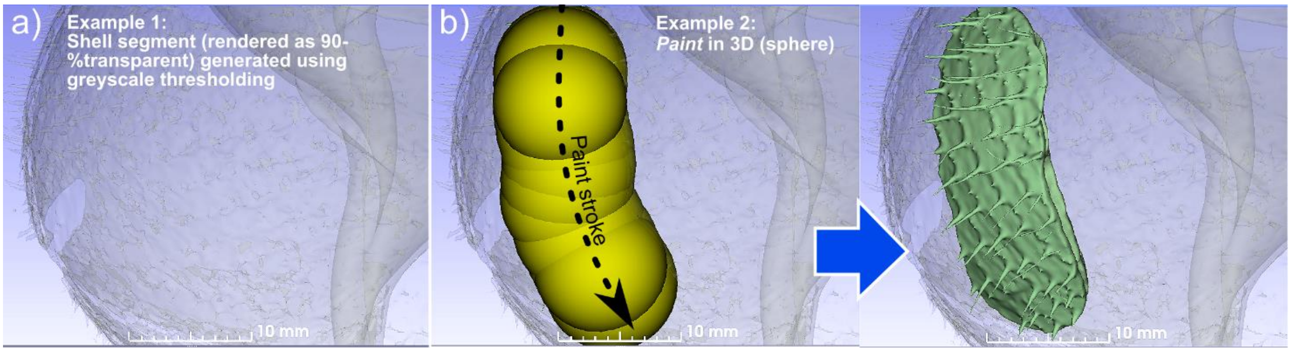

Supplementary methods: figure 2 Examples of thresholding
Examples of thresholding, used to select entire or partial tissue types in *A. kojimai*. a) *Threshold* has been used to select all regions in volume with maximal greyscale values (predominantly calcified shell), which result from high beam attenuation. Segment boundaries are then expanded with the *Margin* tool, under less stringent thresholds, to include surrounding periostracum; b-c) similar results are attainable on a smaller scale, when using thresholded *Paint* in 3D with greyscale ranges suitable for calcified and periostracal shell only but not background. Only shell is selected in active region – as defined by 3D-paint trace in b – with sufficient resolution to depict periostracal bristles (c, overlaid on results from *a*).

3.6.4. Hollow

Create a hollow segment 'skin' from a 'solid' segment, with user-defined wall thicknesses. Used as a start point to create epithelial surfaces of an organ (e.g. cephalopedal-visceral epithelium, some regions of the pallium).

3.6.5. Smoothing

Used to smooth the boundaries of a segment, fill holes of a given pixel-width, or retract extrusions from the segments outline (Supplementary methods: figure 3).

3.6.6. Scissors

Cut away predefined regions of a segment, particularly useful in 3D view (used to remove disconnected blood vessels between image a) and b) in Supplementary methods: figure 3).

3.6.7. Islands

Islands are spatially isolated regions of a segment, which can be removed (Supplementary methods: figure 3), retained exclusively, or split into multiple segments etc. Cavities entirely bound by a segment are also interpreted as internalised islands and can be filled by adding the 'island' to that segment.

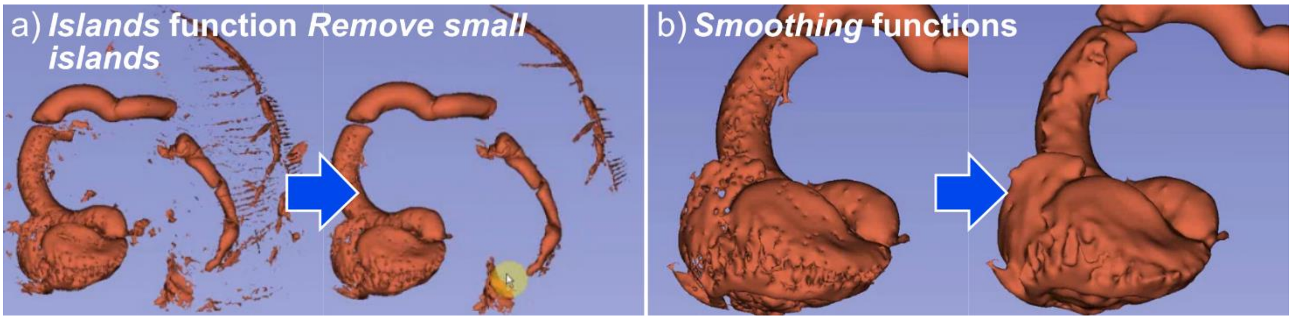

Supplementary methods: figure 3 Cleaning up segments

a) *Remove small islands* function deletes smallest isolated segment regions (based on a maximum number of pixels); b) following the removal of unwanted objects using *scissors* in 3D view, *smoothing* is employed using the *Fill holes* (3-pixel width) function

3.6.8. Logical operators

A suite of operations can be performed on a segment in the context of another segment, such as adding the contents of two segments together, subtracting one from the other, or retaining only those regions common to both (intersect). By creating a new segment and using copy to replicate an existing segment, duplicate segments can be created and edited to retain the original as a back-up.

3.6.9. Draw tube

Draw a solid tube of a given diameter. Very useful for tracking organs that form branching networks for which greyscale values are erratic (e.g. blood vessels with pooled haemolymph and empty spaces).

3.6.10. Grow from seeds / Watershed / Fill between slices / Fast marching

Methods that interpolate (Fill between slices) or extrapolate (Grow from seeds / Watershed / Fast marching) whole segments based on the creation of initial, user-defined 'seed' segments in a number of image slices (Supplementary methods: figure 4). The greater the number of seeding slices the more accurate the results of the interpolation / extrapolation tend to be. Can be updated by additional user input. Fast marching is a little different in that its extent is dependent on the radius from the seeding point and the thresholds inputted.

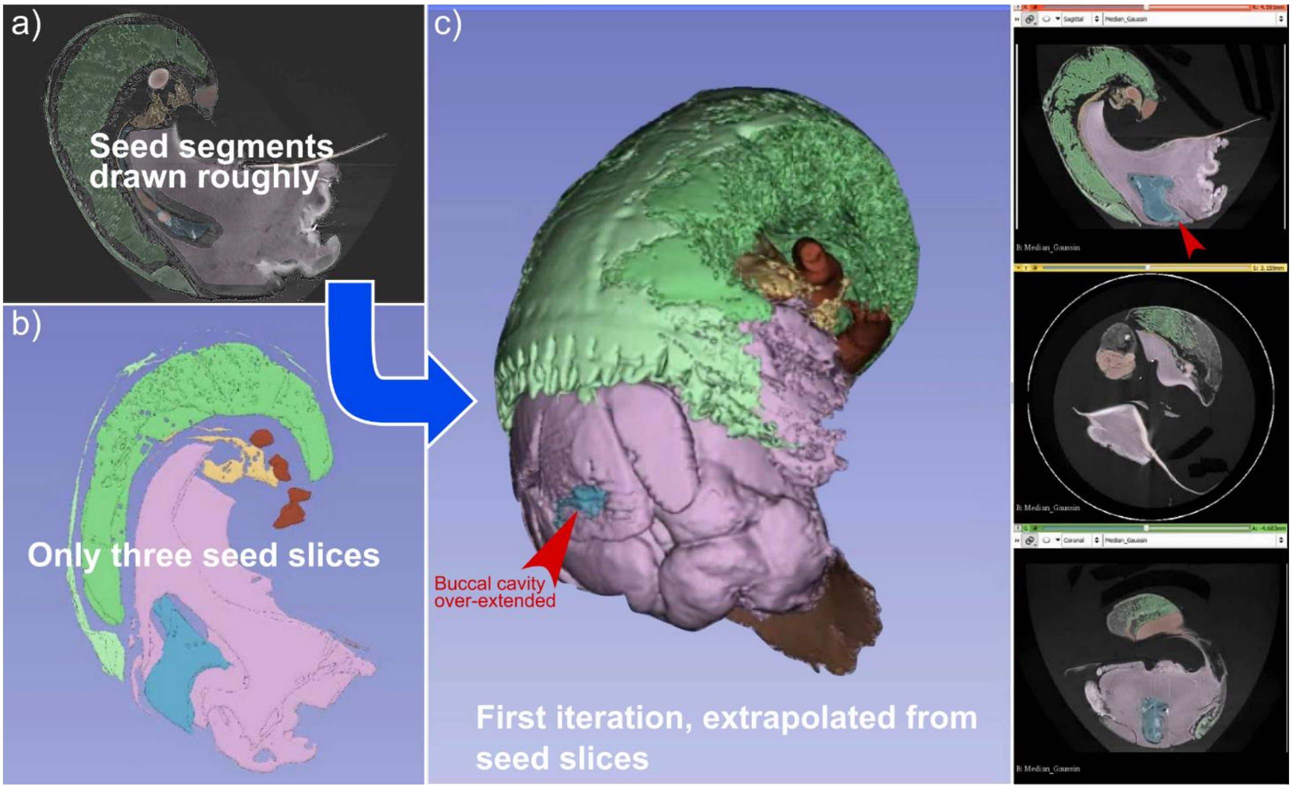

Supplementary methods: figure 4 ***Grow from seeds*** **extrapolation method**
Grow from seeds is a method used to extrapolate full segmentation, based on a number of individual slices for which tissues have been segmented completely. Once initialised, additional slices can be segmented, with the *Grow from seeds* results being updated in real time to reflect the additional user input, typically improving the extrapolated segmentation. Note that *Watershed* is a recently developed method (as of v. 4.10) which performs the same operation, except segment boundaries can be smoothed as part of the process, reducing bleed effects (e.g. red arrowhead, where buccal cavity

segment has extended beyond its natural limits, through the head-foot.). N.B. this example is from a CT scan of a juvenile *Alviniconcha* sp., not presented in the current manuscript.

4. References

1. Doyle J. DNA protocols for plants. In: Hewitt G, Johnson A, Young J, editors. Molecular techniques in taxonomy. Springer, Berlin, Heidelberg; 1991. p. 283–93.

2. Nelson K, Fisher CR. Absence of cospeciation in deep-sea vestimentiferan tube worms and their bacterial endosymbionts. Symbiosis. 2000;28(1):1–15.

3. Folmer O, Black M, Hoeh W, Lutz R, Vrijenhoek R. DNA primers for amplification of mitochondrial cytochrome c oxidase subunit I from diverse metazoan invertebrates. Molecular Marine Biology and Biotechnology. 1994 Oct;3(5):294–9.

4. Shimayama T, Himeno H, Sasuga J, Yokobori S, Ueda T, Watanabe K. The genetic code of a squid mitochondrial gene. In: Nucleic acids symposium series. 1990. p. 73–4.
